# Endothelin-1 mediated vasoconstriction leads to memory impairment and synaptic dysfunction

**DOI:** 10.1038/s41598-021-84258-x

**Published:** 2021-03-01

**Authors:** Latha Diwakar, Ruturaj Gowaikar, Keerthana Chithanathan, Barathan Gnanabharathi, Deepika Singh Tomar, Vijayalakshmi Ravindranath

**Affiliations:** 1 Centre for Neuroscience, Indian Institute of Science, Bangalore, 560012 India; 2 Centre for Brain Research, Indian Institute of Science, C.V. Raman Avenue, Bangalore, 560012 India

**Keywords:** Blood-brain barrier, Cellular neuroscience, Diseases of the nervous system, Learning and memory, Molecular neuroscience, Neuro-vascular interactions, Synaptic plasticity, Biochemistry, Molecular biology, Neuroscience, Physiology

## Abstract

Cerebrovascular lesions seen as white matter hyperintensity in MRI of elderly population caused due to micro-infracts and micro-bleeds contributes to vascular dementia. Such vascular insult caused by impairment in blood flow to specific area in brain involving small vessels can gradually worsen the pathology leading to cognitive deficits. In the present study we developed a transient model of vaso-constriction to study the impact of such pathology by bilateral injection of ET-1 (Endothelin-1; a 21 amino acid vasoconstricting peptide) into lateral ventricles of C57 mice. The impediment in cerebral blood flow decreased CD31 expression in endothelial cells lining the blood vessels around the hippocampal region, leading to memory deficits after 7 days. Activity dependent protein translation, critical for synaptic plasticity was absent in synaptoneurosomes prepared from hippocampal tissue. Further, Akt1- mTOR signaling cascade was downregulated indicating the possible cause for loss of activity dependent protein translation. However, these effects were reversed after 30 days indicating the ephemeral nature of deficits following a single vascular insult. Present study demonstrates that vasoconstriction leading to memory deficit and decline in activity dependent protein translation in hippocampus as a potential molecular mechanism impacting synaptic plasticity.

## Introduction

Dementia is a major public health issue in aging population with increasing life expectancy. It covers a spectrum of disorders including Alzheimer’s Disease (AD), Lewy body dementia and Vascular Dementia (VD), among others and more often vascular component plays a major role in all these conditions. The accurate diagnosis of VD is dependent on neuroimaging, neuropsychological and pathological confirmation^1^. Different types of VD are caused by factors influencing vascular pathologies in tissues at various anatomical location in the brain. Atherosclerotic, micro bleeds and micro-infarcts are some of the vascular insults that cause damage to the brain leading to VD, while occurrence of such events in large vessels has acute and severe outcome seen as hemorrhagic and ischemic stroke^2^.

Vasoconstriction in small vessels could occur periodically over age and this over time contributes to VD. In recent years, cerebral small vessel disease (SVD) is being recognized as a major factor leading to cognitive impairment seen in dementia^3^. Even though there is strong evidence for vascular risk factors as contributors for dementia, it is poorly understood. VD may begin as mild cognitive changes due to blockade of small blood vessels that worsen gradually as a result of multiple minor infarcts, leading to cumulative damage. Patients with multiple micro infarcts as well as repeated occurrence of micro infarcts have high risk of developing dementia^4^. White matter hyperintensities which is caused due to vascular dysfunction during aging correlates with cognitive deficits^5^.

We developed a mouse model of vasoconstriction predominantly in small vessels by intra-cerebroventricular (ICV) injection of endothelin-1 (ET-1) into lateral ventricles, bilaterally. ET-1 is a 21 amino acid endogenous vasoconstrictor peptide and acts through endothelin receptor A or B, a G protein coupled receptors expressed in vascular endothelial cells^6^. ET-1 synthesized by endothelial cells and astrocytes, is altered physiologically during vascular insults to regulate constriction and reperfusion of vessels in patients^7^. Topical administration as well as stereotaxic injections of ET-1 into tissue near middle cerebral artery and sub-cortical injections has been used in many studies to accomplish long lasting vasoconstriction and gradual reperfusion^8^. ET-1 induced vasoconstriction is known to have variability due to response of blood vessels and distribution^9^. We tried to overcome this by injecting into lateral ventricles allowing it to spread through cerebrospinal fluid. In present study ICV injection of ET-1 into lateral ventricles caused vasoconstriction around hippocampal region as observed by decreased CD31 (cluster of differentiation 31) expression, a marker for endothelial cells lining the blood vessels.

## Results

### Decreased expression of CD31 after 3 days ET-1 injection in hippocampal region

**Figure 1:**
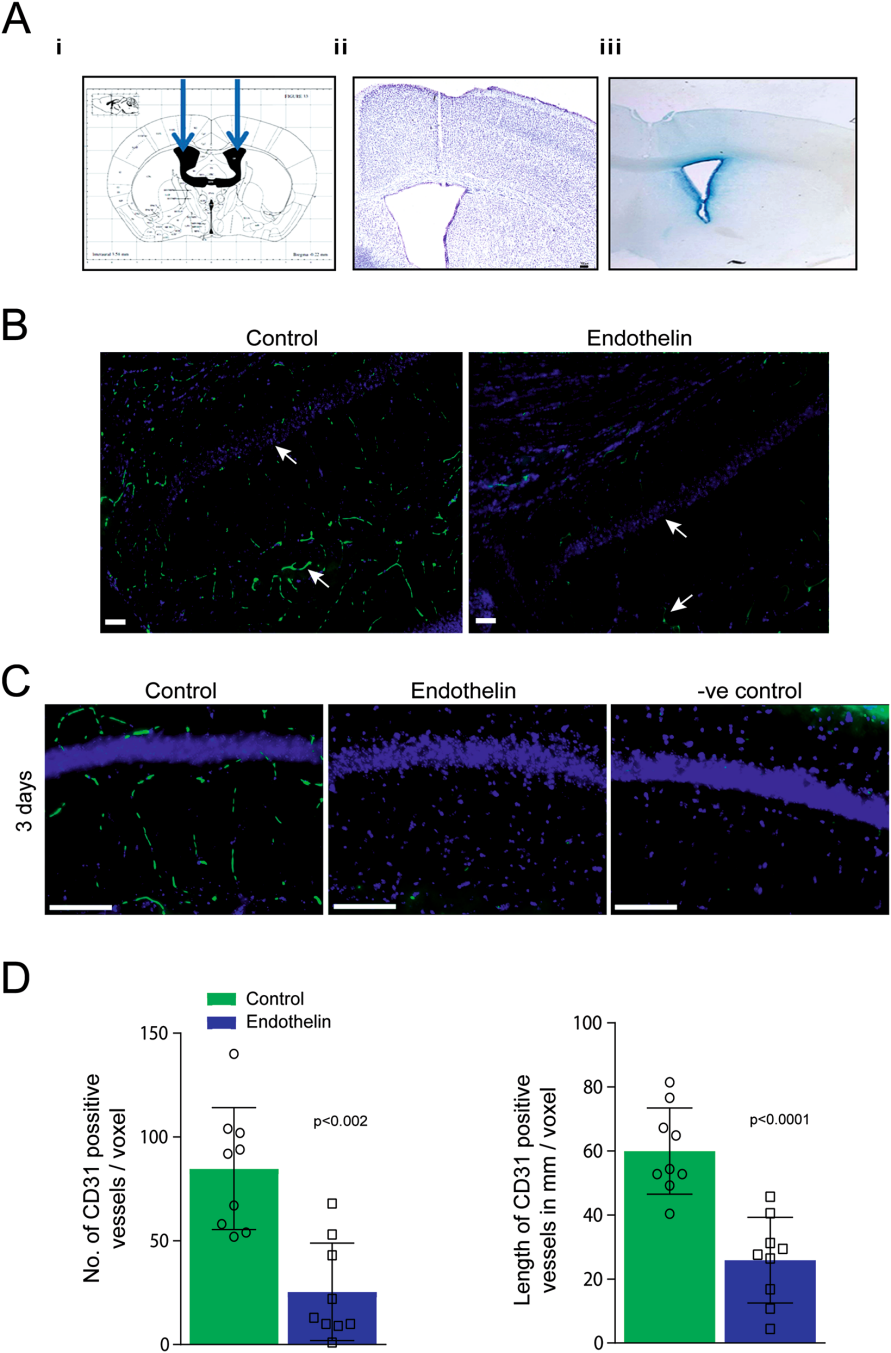
Decreased expression of CD31 after 3 days of bilateral ET-1 injection. (**A**) (i) Shows the site of injection on the atlas of Franklin and Paxinos (1997) of mouse brain depicting the location of cerebral ventricles where ET-1 was injected. (ii) The Nissil staining showing the needle track at the site of injection. (iii) The coronal sections of mouse brain show the dye spread from site injection. (**B**) The expression of CD31 was decreased after 3 days of ET-1 injection in CA1 region of hippocampus compared to vehicle control at ×10 magnification. The arrows show pyramidal layer in CA1 region of hippocampus and micro vessels. (**C**) The images are at 20x magnification of ET-1 injected and vehicle control used for quantitation (-ve control is used to indicate that there was no nonspecific staining). (**D**) The graph showing number and length of CD31 positive blood vessels at 3 days of ET-1 injection. Values are from three sections of hippocampus of control and ET-1 injected mice expressed as Mean ± SD of n = 3 animals with statistical significance.

Mice were injected with Trypan Blue dye according to stereotactic coordinates mentioned in methods (Fig. 1Ai) showed the dye spread across the ventricular space (Fig. 1Aii). Nissl staining of mouse brain after stereotaxic injection shows the needle track clearly leading to the site of injection (Fig. 1Aiii). Immunofluorescence staining in brain sections of C57 mice injected with ET-1 bilaterally into the ventricles showed decreased expression of CD31, a marker for vascular endothelial cells after 3 days of treatment (Fig. 1B,C) and negative control was done to ensure that there was no nonspecific staining. The change in CD31 staining caused by ET-1, was measured in terms of the length and number of CD31 positive vessels. There was significant decrease in the number and length of CD31 positive (Fig. 1D) vessels in ET-1 injected mice compared to vehicle controls. To find out whether loss of CD31 expression lead to Blood Brain Barrier (BBB) leakage we stained sections from early and late time points for blood derived IgG followed by isolectin staining for topography of vessels. It was observed that there was BBB leakage after 3 days of ET-1 injection. However, there was no IgG detected at 30 days of ET-1treatment indicating restoration of BBB (Sup. Fig. 1). Further, ET-1 injection into lateral ventricles did not cause apparent neuronal loss in hippocampus as observed by NeuN staining (Sup. Fig. 2).

### Vasoconstriction leads to learning and memory deficits 7 days after ET-1 injection

**Figure 2:**
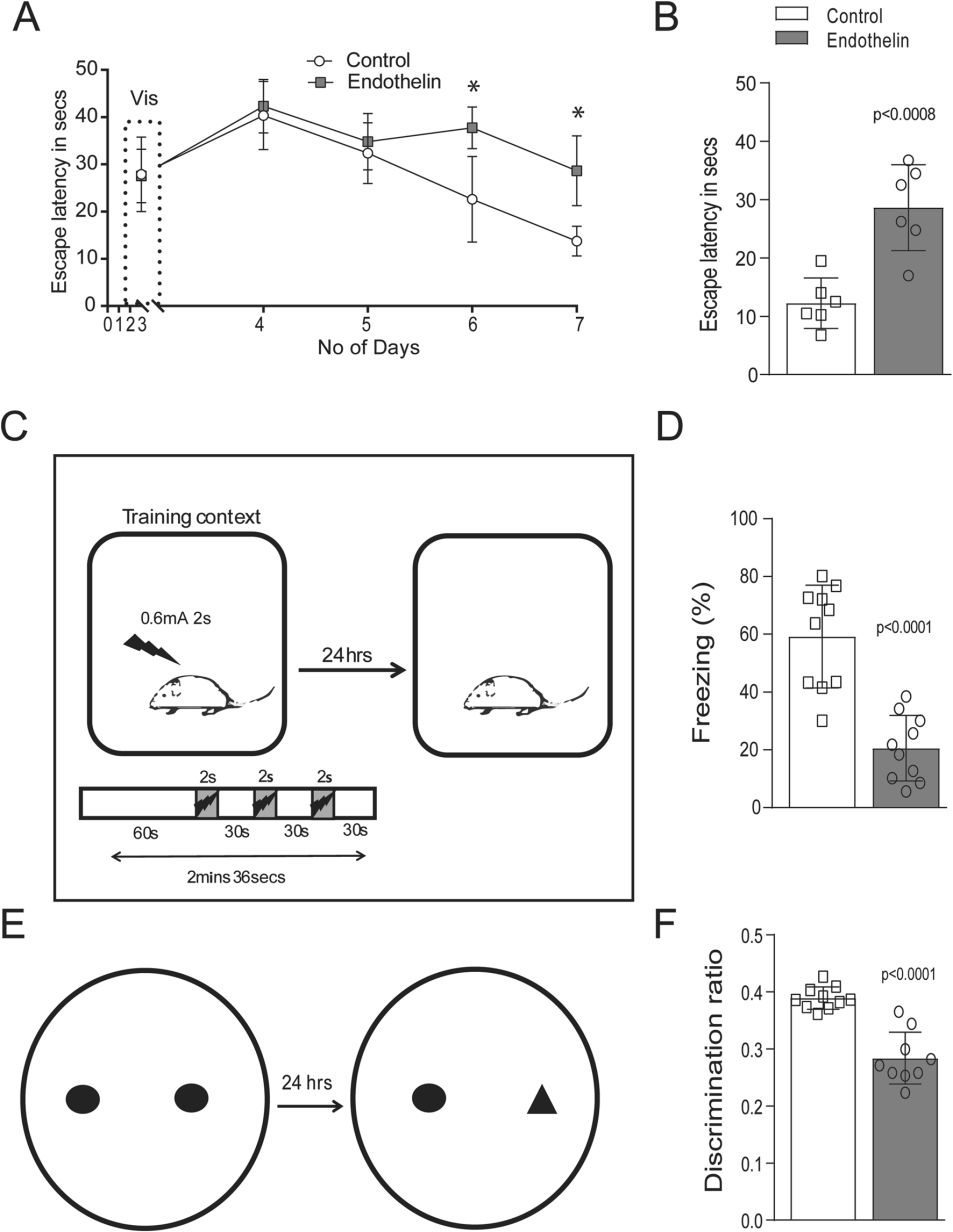
Evaluation of spatial and associative memory deficits. (**A**) The graph shows spatial memory deficit in ET-1 injected mice, on day 3 (visible platform) there is no difference between the groups. From day 4–7 (hidden platform) there was significant difference in escape latency suggesting ET-1 treated mice performed less compared to controls. (**B**) The bar graph indicates escape latency performance after 7 days of ET-1 treatment, mice took more time to find the platform which is expressed as Mean ± SD of n = 6 animals. (**C**) Representative illustration of context fear conditioning test set up. Both the groups were given 0.6 mA shock after 6 days of ET-1, recall was done after 24 h at 7 days of ET-1. (**D**) There was significant decrease in percentage of freezing after 7 days ET-1 injection. Values are Mean ± SD of n = 10 animals. (**E**) First panel depicts the illustration of novel object recognition task. (**F**) The graph indicates defective memory performance in the test phase after 7 days of ET-1 injection expressed as Mean ± SD of discrimination ratio of n = 10 animals.

We evaluated the consequence of reduced blood flow on learning and memory paradigm in these animals. We trained mice on Morris water maze after 2 days of injection to assess their spatial memory (Fig. 2A). On day 6 and 7 of the Morris water maze trials, ET-1injected mice showed significantly increased escape latency time compared to saline treated mice (Fig. 2B). We performed contextual fear conditioning test (cFc) on day 6 after ET-1 treatment, mice were given foot shock and the recall in the same context was examined after 24 h (Fig. 2C). We observed a dramatic decrease in freezing response 7 days after ET-1 injection (Fig. 2D) indicating a failure in the recall to the context. The cFc recall deficit observed in ET-1 injected mice was similar to that seen in 9-month-old male APP/PS1 (APPswe/PS1ΔE9) mouse model of AD (Suppl. Fig. 3). We performed novel object recognition test to assess episodic memory, which also represents proper functioning of hippocampal circuitry function in ET-1 infused and saline controls. The ability of mice to recognize novel object verses familiar object during the test phase was assessed (Fig. 2E). ET-1 injected mice showed considerable decrease in exploration time with the novel object as indicated by discrimination ratio after 7 days (Fig. 2F). Thus, the tests performed to assess spatial and associative memory showed impairment after 7 days of ET-1 injection demonstrating the vasoconstriction caused by ET-1 is leading to learning and memory deficits.

The gait parameters were measured for mice injected unilaterally with ET-1 in left hemisphere of brain by appraising the footprint patterns. The rear and front paws were inked with nontoxic paint, allowed walk through a tunnel with a strip of paper on the floor. The resulting foot patterns were assessed quantitatively to 5 measurements for stride length and gait (stance) width. There was no change in step length or in gait width compared to controls (Sup Fig. 4A and B).

We measured the motor performance ability of mice after ET-1 injection on accelerating rotarod for 15 days every alternate day after 2 days of surgery. There was no difference in the performance of rotarod between ET-1 injected and saline on all the days (Sup Fig. 4C). The mice stayed on the accelerating rotarod for about 150 s indicating there was no motor deficit due to unilateral injection of ET-1.

### Activation of microglia and increase in Iba-1 expression after ET-1 injection

**Figure 3:**
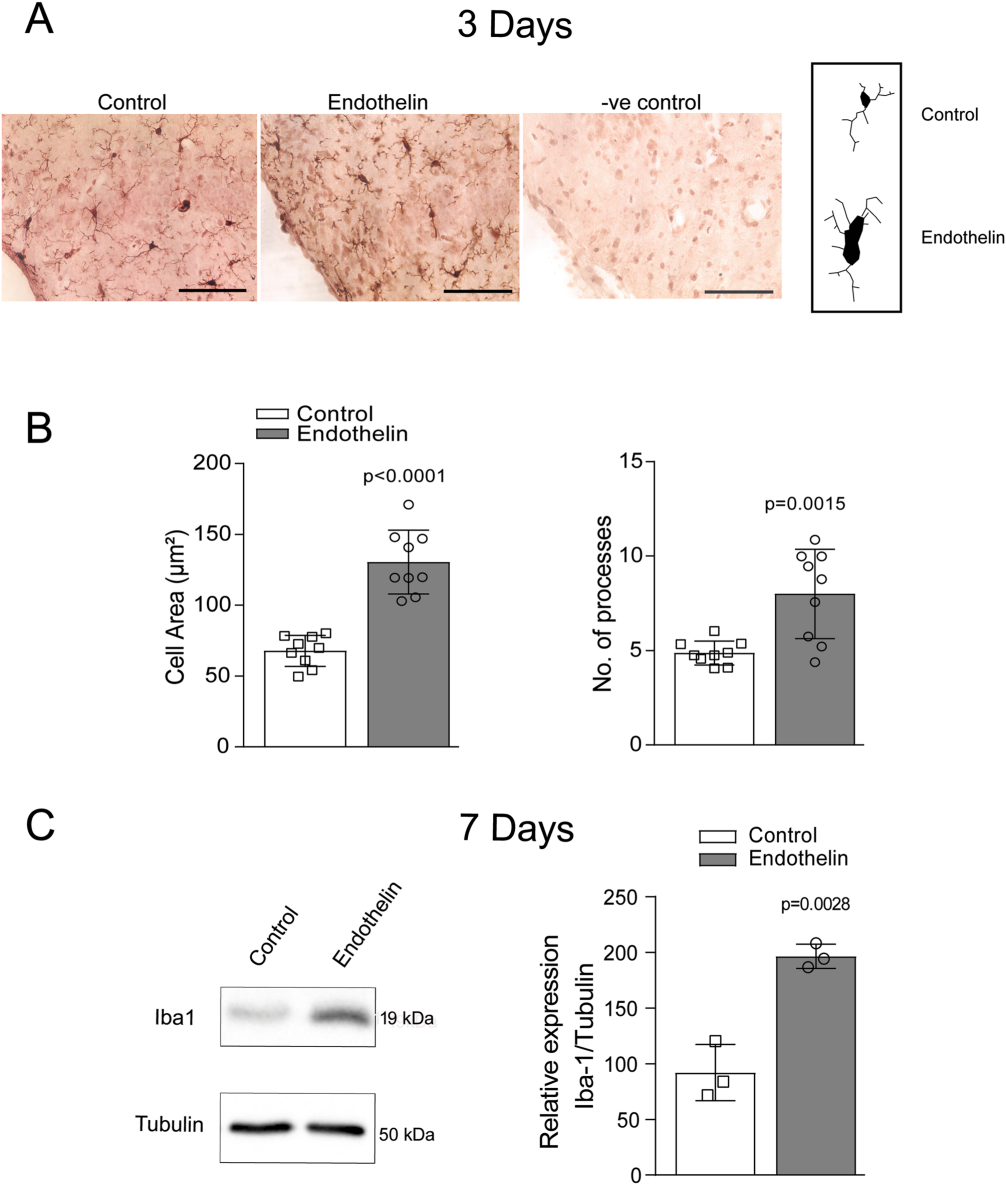
Increased expression of Iba-1 in CA1 showing microglial activation near the ventricles. (**A**) ET-1 treatment stimulated microglial activation after 3 days around the ventricles. (**B**) The graph shows significant increase in cell area and number of process in activated microglia of ET-1 treated mice compared to vehicle controls (Three sections from each animal was selected for quantification. n = 3). (**C**) The immunoblot showed increased expression of Iba-1 protein after 7 days of ET-1 treatment in hippocampal lysate. The bar graph shows significant increase in relative expression of Iba-1 in ET-1 treated mice compared to vehicle control (n = 3).

We performed immunostaining for microglial marker Iba-1 (ionized calcium binding adaptor molecule 1) in mouse brain hippocampus (Fig. 3A). We observed a significant increase in area of cell body of microglia. The number of processes increased in mice injected with ET-1 compared to saline (Fig. 3B). The increase in number of processes and area of cell body in ET-1 injected mice potentially indicated the phagocytic nature of activated microglia after 3 days of ET-1 injection. We also considered whether inflammation persists until 7 days when we saw the behavioral deficits and found significantly increased expression of Iba-1 (Fig. 3C). These results demonstrated inflammatory changes following ICV injection of ET-1.

### Activity dependent protein translation is curtailed in synaptoneurosomes from hippocampus of mice injected with ET-1

**Figure 4:**
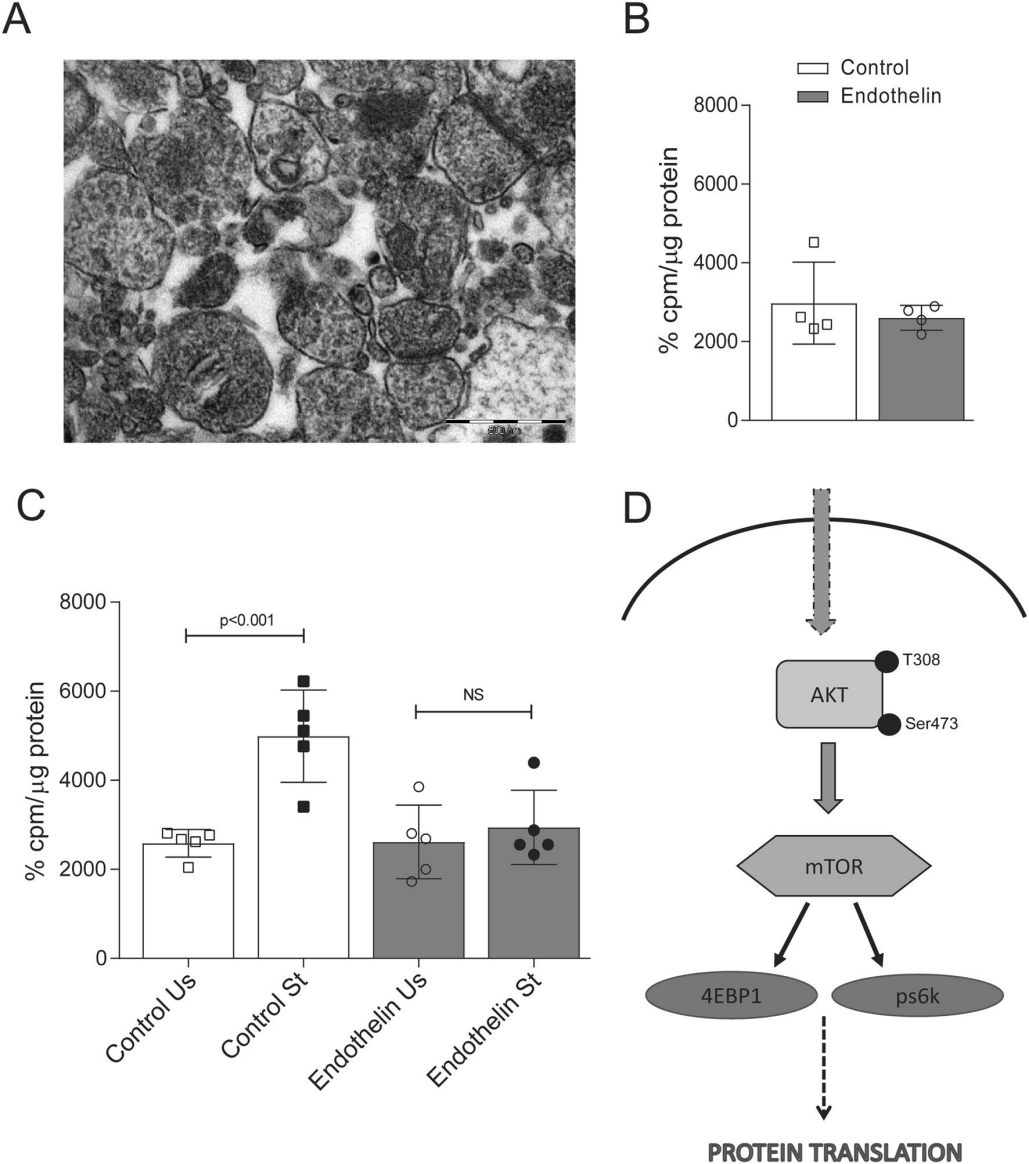
Local activity-dependent protein translation at the synapse is diminished in ET-1 treated mice. (**A**) Electron micrograph of synaptoneurosomes indicating postsynaptic density and vesicles in presynaptic terminal. (**B**) Protein translation measured as S^35^-methionine incorporation in PNS fraction of hippocampus was unaffected in control and ET-1 treated mice. (**C**) Synaptoneurosomes prepared from hippocampus of control and ET-1 treated mice were subjected to protein translation after KCl stimulation was measured as S^35^-methionine incorporation. Stimulation of protein translation in synaptoneurosomes in the presence of KCl was abolished in ET-1 treated mice after 7 day of ET-1 treatment. Data are represented as Mean ± SD (n = 5) and values are significantly different from corresponding controls. (**D**) The graphical representation shows different players involved in Akt-mTOR pathway leading to protein translation.

Activity dependent protein translation is important to maintain synaptic plasticity. Therefore, to know the effect on behavioral dysfunction in present study we measured activity dependent protein translation in synaptoneurosomes isolated from hippocampus of ET-1 injected mice. The structural integrity of synaptoneurosomes was evaluated by electron micrograph before S^35^ (Sulphur 35) incorporation assay (Fig. 4A). Total protein translation as assessed by S^35^ incorporation did not change in the hippocampal post-nuclear supernatant (Fig. 4B) indicating that total protein translation was unaffected by ET-1. Activity dependent protein translation was assessed by stimulating hippocampal synaptoneurosomes with 50 mM KCl (potassium chloride) and measuring newly synthesized protein through incorporation of S^35^ methionine.

There was increased incorporation of S^35^ methionine upon stimulation of hippocampal synaptoneurosomes with KCl indicating higher protein translation at the synapse. However, S^35^ methionine incorporation was unchanged in mice 7 days after injection with ET-1 (Fig. 4C) indicating that activity dependent protein translation is affected at the synapse following vasoconstriction. Since activity dependent protein translation is critical for synaptic plasticity these changes may be affecting learning and memory deficits seen after 7 days of ET-1 injection.

### Akt-1 and GSK phosphorylation are down regulated during the transient ischemia caused by ET-1 injection

**Figure 5:**
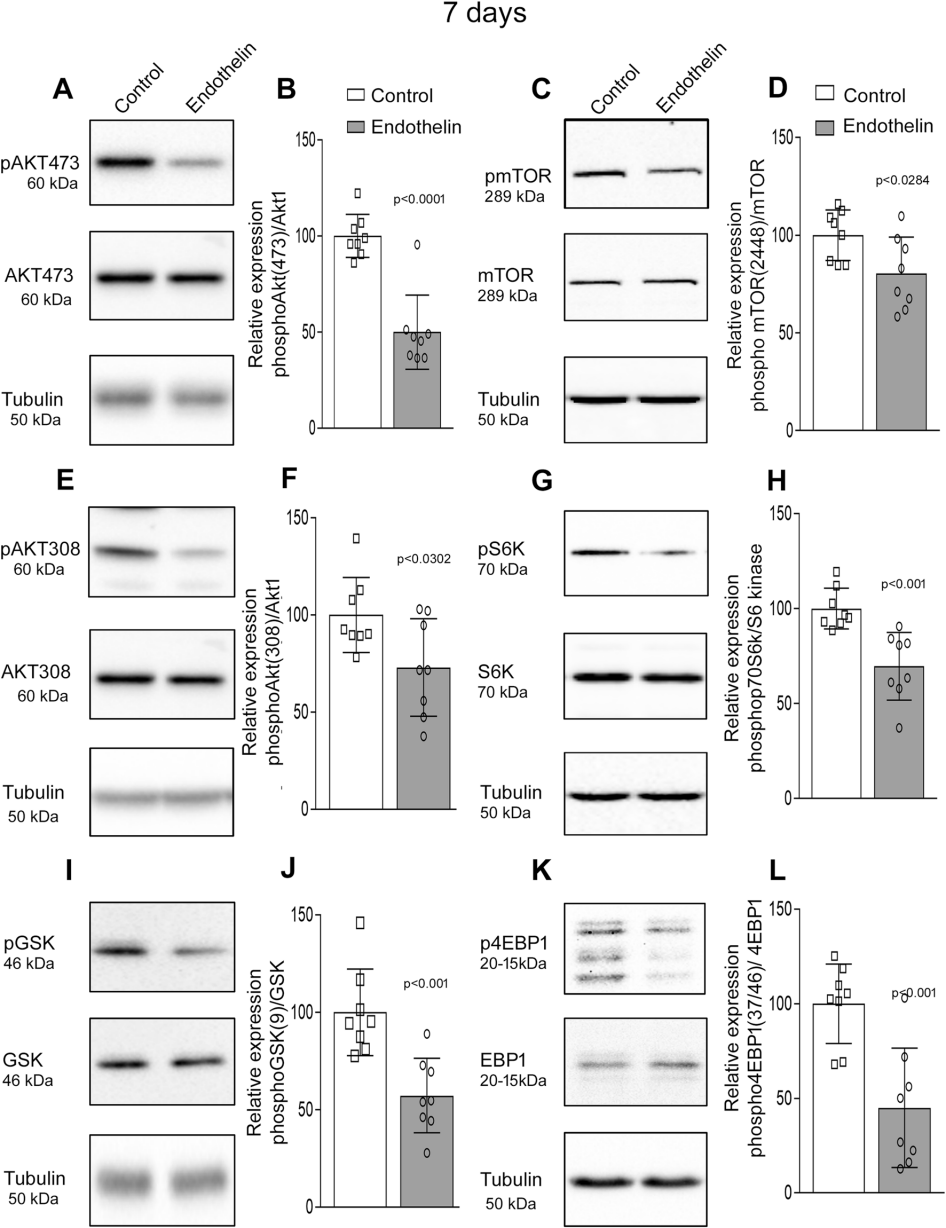
Impairment in Akt1-mToR pathway phosphorylation in hippocampal lysate of bilateral ET-1 injected mice. (**A**) The levels phosphorylated forms of Akt1 Ser473 was decreased in ET-1 injected mice. However, there was no difference in total Akt1 form between ET-1 injected and vehicle control. (**B**) The graph represents the relative expression phospho Ser473 to total Akt1 and values are Mean ± SD, n = 8 animals. (**E**) Phosphorylation of Thr308, which are critical for kinase activity was decreased after 7 days of ET-1 treatment. (**F**) Shows the data of relative expression phospho Thr308 to total Akt1 and values are Mean ± SD, n = 8 animals. (**I**) Further phosphorylation of GSK was also low after 7 days of ET-1 treatment compared to total GSK. (**J**) Graph shows the values of Mean ± SD of n = 8 animals, values are significantly different from corresponding controls. (**C**) There was decrease in levels of phosphorylated mTOR2448 in ET-1 treated mice. (**D**) The graph represents relative expression phosphomTOR to total mTOR, values are Mean ± SD of n = 8 animals. (**G**) The downstream molecule of mTOR, pS6K at Thr389 was low in hippocampal lysate of bilateral ET-1 injected mice after 7 days of ET-1. (**H**) The data shows significant decrease between ET-1 treated and vehicle control. (**K**) The blot showed decreased expression of p4EBP at Thr46/47 after 7 days of ET-1 treatment. (**L**) The graph represents significant difference in relative expression of p4EBP to total 4EBP in ET-1 treated mice after 7 days.

Protein translation is dependent on Akt1-mTOR (RAC-alpha serine/threonine- protein kinase- mechanistic target of rapamycin) signaling in brain. Studies have shown that this pathway is very important in regulating translation and our earlier studies in AD mice have shown that Akt-mTOR pathway is critical regulator of activity dependent protein translation at the synapse^10^ (Fig. 4D). We examined the Akt-mTOR signaling cascade in the hippocampal lysate 7 days after injection of ET-1 or saline. Akt1 is activated by phosphorylation at Thr308 (Threonine) and then at Ser473 (Serine). We saw decreased phosphorylation of both Akt1 at Thr308 and Ser473 in 7 days after ET-1 injection (Fig. 5A,B,E,F). This was in concurrence with changes of behavioral deficit and decrease in activity dependent translation at the synapse 7 days after ET-1 treatment. Further we also observed consequent decrease in pGSK (phosphorylated glycogen synthase kinase), which is downstream of pAkt 7 days after ET-1 injection (Fig. 5I,J). Phosphorylated Akt1 indirectly activates mTOR by phosphorylating TSC1/2 (tuberous sclerosis protein complex 1/2) complex, which is a negative regulator of mTOR there by activating the pathway to phosphorylate 4EBP1 (eukaryotic initiation factor 4E- binding protein-1) at Thr46/47, S6K (p70 Ribosomal S6 kinase) at Thr389 both are important in initiating protein translation downstream (Fig. 4D). The pmTOR (Ser2448) levels were decreased in hippocampal lysate after 7 days of ET-1 (Fig. 5C,D). Further, we observed that there was simultaneous decrease in downstream effector molecules pS6K (Fig. 5G,H) and p4EBP1 (Fig. 5K,L). mTOR phosphorylation is also known to cause energy deficiency leading to autophagy^11^, however in our studies we did not observe any changes in LC3b I/II ratio (Sup. Figure 5). These results demonstrate that Akt1-mTOR signaling pathway, which is important in maintaining protein translation particularly at the synapse is downregulated thus impeding synaptic plasticity.

### Memory impairment and synaptic function caused by a single dose of ET-1 is reversed in 30 days

No behavioral deficits were observed 30 days after ET-1 thus indicating that the learning and memory deficits observed at 7 days following small vessel vasoconstriction were reversed completely. This reversal was seen in novel object recognition test after 30 days ET-1 (Sup Fig. 6A) and recall after contextual fear conditioning (Sup Fig. 6B). Immunostaining done for CD31 expression in sections from the above set of mice showed no difference in number and length of CD31 positive vessels between saline and ET-1 (Sup Fig. 6C and D). BBB leakage was not seen after 30 days of ET-1 injection in concordance with reversal of behavioral deficits (Sup. Fig. 1). Further, activity dependent protein translation in hippocampal synaptoneurosomes from mice treated with ET-1 for 30 days was similar to vehicle controls (Sup Fig. 6E). These results indicate that vascular homeostasis is reestablished within 30 days after ET-1treatment restored Akt-mTOR signaling players Akt-1 (Sup Fig. 7 A, B, E & F), GSK (Sup Fig. 7 I & J), mTOR (Sup Fig. 7 C & D) and downstream molecules like S6K as well as 4EBP1 leading to reversal of the memory deficits (Sup Fig. 7 G, H, K & L). The microglial activation was also reversed by 30 days of ET-1 treatment (Sup Fig. 8 A, B & C).

## Discussion

We have developed a mouse model of vasoconstriction involving predominantly small vessels using a single, bilateral injection of ET-1 into the lateral ventricles, which decreased CD31 (a marker for endothelial cells) levels in the small vessels around the hippocampal region (Fig. 1B). This leads to spatial and associative memory deficits but no motor dysfunction. Activity dependent protein translation at the synapse is essential for synaptic plasticity and this was compromised in the hippocampus 7 days after ET-1 treatment when memory deficits were seen. Akt-mTOR pathway, which is critical for activity dependent protein translation was downregulated. However, these deficits reversed 30 days after ET-1 injection indicating that the damage caused by vasoconstriction was repaired with abrogation of behavioral deficits and restoration of integrity of BBB, activity dependent protein translation and Akt-mTOR signaling.

ET-1 is an endogenously secreted vasoconstricting peptide with considerable long-lasting action for days^12^. Earlier reports have shown that direct injection of ET-1 into the brain (sub-cortical, intracortical or hippocampal) produces expected behavioral deficits that last up to few days. In this study, we have attempted to generate a model of vasoconstriction akin to that seen after a single transient ischemic attack, wherein behavioral and biochemical deficits are observed initially but are completely reversed after 30 days.

ET-1 injection (2 µg) directly into rat brain tissue (cortex, striatum and hippocampus etc.) produces irreversible, focal lesion resulting in permanent behavioral deficits in locomotion and or memory depending on site of injection^13–15^. Intracerebral injection of lower doses of ET-1 (0.5–1 μg) into mouse brain cortex produces infarct induced lesion that are largely resolved by 3 days^16^. Intraventricular injection of ET-1 (9 pmol) in rats has shown metabolic stimulation, however there was no ischemic effect on neurons or glia^17^. In present study we injected ET-1 (2 µg per hemisphere, that is 4 µg in total per mouse) into the lateral ventricles, which is much higher dose considering the body weight of mouse is 25 gm vs 250gm, the average body weight of rat. Earlier studies have reported that injecting higher doses of ET-1 (4 µg/ mouse) into the cortex results in mortality^18^. However, in present study since ET-1 was injected into the ventricles its concentration was presumably diluted as it diffused into the surrounding brain tissue including hippocampus. CSF (cerebrospinal fluid) subsequently flows through the ventricular system of the brain, which consists of the two lateral ventricles and the third ventricle, which finally connects to the subarachnoid space. CSF is mainly secreted by specialized vascular structures, the choroid plexuses in the ventricle^19,20^. It has been shown that active transport can be bi-directional over the epithelial cells of the choroid plexus^21^. The direction of flow, the anatomical structures involved, and the driving forces that are involved in interstitial fluid and CSF flow is controversial^22,23^. Thus, ET-1 injection into ventricles could also have effect on CSF flow and choroid plexuses and this needs to be investigated further. Thus, our mouse model of vasoconstriction enabled us to deliver sufficient dose of ET-1 to bring about adequate vasoconstriction in brain regions while there was no lethality.

We then explored the molecular mechanisms that could underly the behavioral deficits seen in mice expose to ET-1. Synaptic plasticity is an important attribute for learning and memory and activity dependent protein translation at the synapse is essential for synaptic plasticity. We therefore assayed incorporation of S^35^ methionine in synaptoneurosomes from hippocampus (7 days post ET-1) after they were stimulated by 50 mM KCl. While S^35^ methionine incorporation increased significantly in vehicle injected mice upon stimulation with KCl this was not observed in ET-1 treated mice indicating that activity dependent protein translation was non-existent following ET-1 treatment.

We observed ET-1 induced vasoconstriction downregulated both the phosphorylated forms of Akt1 (Ser473 and Thr308) in hippocampal lysate leading to compromised protein translation in the synaptoneurosomes. Moreover, there was loss of pmTOR and its downstream substrates, pS6k and p4EBP1 in hippocampal lysate of ET-1 injected mice at 7 days (Fig. 5). Earlier studies from our lab have shown that activity dependent protein translation is absent in synaptoneurosomes because of loss of Akt1 kinase activity leading to downregulation in Akt-mTOR pathway in very early (1-month-old) in AD mouse model (APP/PS1)^10^. Studies on amyloid-β peptide (Aβ) interaction with the vasculature have shown that ET-1 mediates oxidative stress through the receptor for advanced glycosylation end products (RAGE), leading to reduction in blood flow in AD mice^24^. Further these Aβ-induced perturbations in cerebral vessels, neurons, and microglia in AD can be effectively controlled by RAGE blocker^25^. Thus, it would be important to study the importance of ET-1 induced vasoconstriction in presence of Aβ.

Our results indicate for the first time the commonality between vasoconstriction in brain and early AD in downstream pathways that drive the behavioral abnormalities. However, unlike in AD both the behavioral and molecular perturbations were reversed by 30 days after single injection of ET-1 indicating that these phenomena are reversible much like that seen in patients with transient ischemic insults.

Studies have shown that in neurodegenerative disorders including AD patients, show microvasculature dysfunction and defective BBB^26^. Microvascular defects are known to diminish the blood flow causing hindrance in clearing toxic molecules from brain leading to their accumulation, which is observed in conditions like cerebral amyloid angiopathy. Therefore, in addition to brain endothelium, vascular smooth muscle cells, pericytes, astrocytes and activated microglia are important in cerebral vascular function^27,28^.

More recently the important role of pericyte in regulating vasculature specially during regeneration has been described. This is considered to occur through pleiotrophin, which is secreted by the pericytes^29^. In the present study we have observed the regeneration of vessels and restoration of the behavior and molecular dysregulation. It is yet to be determined if pericytes, through secretion of the trophic factor, pleiotrophin play a role in the regenerative process. It is important to understand the molecular under pinning of regeneration of vessels since it offers potential therapeutic avenues for people with vascular dysfunction (infract and micro bleeds) seen as white matter hyperintensities in MRI.

Vascular dementia contributes majorly to the total dementia burden and is usually caused by cerebrovascular events involving small vessels^30^. The molecular etiology of such vasoconstriction needs to be understood better. In this study we have developed a reversible model of small vessel vasoconstriction that presents the dementia phenotype for a short period (7 days) and is reversed 30 days. This model gives us an opportunity to identify the molecular underpinnings that lead to vascular dementia phenotype, which we have determined as loss of activity dependent protein translation and downregulation of Akt-mTOR pathway thus demonstrating the similarities in early molecular events between vascular dementia and early AD.

## Materials and methods

### Animals

C57BL/6 mice used in all the experiments were maintained in central animal facility, Indian Institute of Science in controlled light and temperature. Mice were maintained in 12-h dark and light cycle, had access to food pellets and ad libitum. Only male mice aged 3.5–4.5 months of age (25–30 g) were used in the study. Efforts were made to minimize the number of animals used and ensure minimal sufferings. All the animal experiments were designed and followed according to Committee for the Purpose of Control and Supervision of Experiments on Animals (CPCSEA), Government of India, which is in compliance with the ARRIVE guidelines. Institutional animal ethics approval from Indian Institute of Science, Bengaluru, India was obtained for all the animal experimentation (CAF/Ethics/528/2016).

### Materials

Primary antibodies against pAkt1 (Ser473, Thr308), Akt1, pGSK3β (Ser9), p mTOR (Ser2448), mTOR, pS6K (Thr389), S6K, p4EBP1 (Thr46/47), 4EBP1 and LC3b I/II were purchased from Cell Signaling Technologies, Denvers, MA, USA. Anti-CD31 anti-Iba-1, anti-Abeta and anti NeuN antibodies are from Abcam, and anti-β-tubulin antibody was from Sigma, St. Louis, MO, USA. Tetramethylbenzidine (TMB) substrate kit and secondary antibodies; anti-rabbit and anti-mouse conjugated with horse-radish peroxidase (HRP) were purchased from Vector Laboratories, Burlingame, CA, USA. Endothelin-1 was purchased from Millipore. Isolectin dye was purchased from Life science technologies. All other chemicals and reagents used were of analytical grade and obtained from either Sigma-Aldrich or Merck.

### Vasoconstriction induced by ET-1

Animals were anesthetized with ketamine and xylazine (4:1), positioned on a stereotaxic frame (Stoelting Co., Wood Dale, IL, USA). We injected ET-1 bilaterally into the ventricles at following coordinates, 0.02 mm anterior, 1.0 mm lateral of bregma on either side and 0.22 mm in depth. We infused 2 µg ET-1 in 2 µl on each side over 20 min and the needle was withdrawn after 5 min of injection to prevent the backflow. In some experiments (for gait and rotarod test) animals were injected only on one side (left) to test the effect of ET-1 injection on motor function. Control animals were injected with saline at same flow rate and time. Animals were maintained at 37.0 ± 0.5 °C with a heating pad throughout the experimental and recovery periods.

### Brain sectioning and Immunohistochemistry

After 3 days of ET-1 injection mice were anesthetized, perfused transcardially with PBS followed by 4% paraformaldehyde (w/v). Brains were dissected out, post fixed in 4% paraformaldehyde (w/v) overnight and transferred to 30% sucrose (w/v) in PBS. Cryosections of 20 µm were cut and immunohistochemistry was performed according to established protocol using anti CD31 (Abcam) or anti Iba-1 antibody (Wako chemicals, Richmond, VA, USA)^31^. We selected sections from dorsal, middle and ventral hippocampus for quantitation, which was done in a double-blind manner. Stained brain sections were imaged in fluorescence or bright field microscope, as appropriate. The CD31 expression of blood vessels were captured in two fields on either side of the ventricles under 20 × objective from three sections of hippocampus from rostral to caudal per mouse. Image J software was used to count the numbers and length of CD31 positive blood vessels. Quantification was averaged and expressed as number or length of CD31 positive vessels per section.

Similarly, we took higher magnification images (40x) in all the three hippocampal sections from dorsal, middle, and ventral regions of mice injected with ET-1 and saline. Further, we quantified the cell volume and number of processes in microglia to show the activation using Neuroleucida software. Hippocampal sections were incubated with mouse IgG followed by Isolectin to check for Blood brain barrier leakage^29,32^ and with neuN antibody to check for neuronal lss or with Aβ antibody for plaques according to earlier protocols^31,33^. The sections were imaged under fluorescence or bright field microcopy.

### Behavioral parameters

The animals were randomized after treatment and all tests were conducted by an experimenter blinded to the treatment. All animals were handled for 3 days prior to testing to avoid stress.

#### Morris water maze

Spatial memory was assessed using Morris water maze test in saline and ET-1 injected mice (10 mice per group) by standard hidden platform training^34^. The mice were subjected to spatial task after 2 days of injection. A circular pool (1.1 m diameter) was filled with water (kept at 19 ± 1 °C), and a transparent platform (15 cm in diameter) was placed in one quadrant of the pool. The water was made cloudy with nontoxic tempera paint, and the room was lit optimally with visuospatial cues on the curtain covering the pool. The test was performed over 7 days and consisted of cued task with visible platform at day 1, in which the platform was kept 1 cm above the water level. The mice were left to swim from all the four quadrant and underwent four trials of 60 s each to find the platform. If the mice fail to reach the platform by 60 s, they were guided and allowed to stay on platform for 15 s. From second day platform was submerged 1 cm below the water surface and mice were subjected to 4 trials as above to find the platform. The behavior was recorded on video camera and latency period was calculated manually. The animals were allowed to dry under a heat lamp after each trial to avoid hypothermia, and all experiments were started at the same time each day.

#### Contextual fear conditioning test

Animals were single housed after stereotaxic injection (no injection in case of APP/PS1 mice) and handled for 3 min three days before the experiment. The experiment was performed in a standard soundproof chamber with stainless-steel grid floor connected to solid state scrambler. The scrambler is connected to electronic shocker controlled through computer program (Coulbourn instruments, PA, USA). A digital camera is fixed on top to record the behavior videos. Animals could explore square contextual fear conditioning (cFC) training context for 1 min and then received 3-foot shocks (0.6 mA at 30 s interval) after 6 days or 29 days of ET-1 injection. The square training context was cleaned with 70% alcohol (vol/vol) between each session with the animals. Contextual fear memory to the training context was assessed after 24 h of fear conditioning by bringing the animals to same chamber. The fear memory was evaluated by scoring freezing behavior expect for respiration.

#### Novel object recognition test

Object recognition paradigm was used to examine episodic-like memory perse. The animals were handled for 3 min for 3 days before the task and kept in testing room 1 h before to avoid stress. The open field consisting of circular container with diameter 60 cm and depth of 28 cm, animals were allowed explore the context for 5 min one day before sample phase. In sample phase, two identical objects were presented, and animals could explore for 10 min. In test phase, one object was the same while the other object was novel, and animals explored for 10 min. The test was performed after 5–7 days or 27–30 days of ET-1 injection. The results are expressed as discrimination ratio of time for novel object verses familiar object^35^.

### Gait analysis

Animals injected with ET-1 unilaterally in left side of hemisphere were subjected to gait analysis. The animal’s footprints were taken a day before surgery and every alternate day for 15 days after 2 days of ET-1injection according earlier literature with little modification^36^. The hind and front feet were coated with nontoxic paint, animals were allowed to walk through a dark 50 cm-long, 9 cm-wide, 10 cm-high tunnel. Then the footprint patterns made on the paper lining the floor of the tunnel were joined with pencil and distance were measured for each step length of forward movement. Average distance for each foot was calculated considering five steps for each animal. To measure gait width, average lateral distance between opposite left and right steps of front and hind feet was determined by measuring the perpendicular distance of a given step to a line connecting to its opposite preceding and succeeding steps.

### Accelerating rotating rod test

The rotating rod apparatus was used to measure the ability of mice to improve motor skill performance with training. Mice were trained for three consecutive days, each session of three trials with a gap of 15 min between each trial to avoid fatigue and exhaustion. Mice were placed on the rod for three trials per day for alternate days after 2 days of single dose of unilateral ET-1 injection and trial was done for span of 15 days. Each trial lasted a maximum of 150 s, during which time the rotating rod underwent a linear acceleration from 5 to 25 rpm with each step lasting for 30 s for total of 150 s. Animals were scored for their latency to fall (in seconds) for each trial. If the animal did not fall, then criterion for 150 s was recorded. The average of three trials in each session was calculated for each animal and results were analyzed statistically compared to controls.

### Activity dependent protein translation assay

Synaptoneurosomes from mouse hippocampal tissues were prepared according to earlier protocol^10,37^. Tissue was homogenized in translation buffer consisting 118 mM NaCl (Sodium Chloride), 4.7 mM KCl, 1.2 mM MgSO_4_ (Magnesium Sulfate), 2.5 mM CaCl_2_ (Calcium Chloride), 1.53 mM KH_2_PO_4_ (Potassium dihydrogen phosphate), 212.7 mM glucose and 1 mM 1,4-dithiothreitol (DTT) (pH7.4) with protease and phosphatase inhibitors, 200 µg/ml chloromphenicol and 30 U/ml RNAse inhibitor. Homogenate was filtered sequentially through two 100 µm and one 10 µm membrane filters (Millipore). The filtrate obtained was then centrifuged at 1500*g* at 4 °C for 10 min, the pellet containing synaptoneurosomes was resuspended in translation buffer. Synaptoneurosomes was stimulated by incubating with 50 mM KCl at 37 °C for 15 min in presence of 50 µCi S^35^-L-methionine. Unstimulted synaptoneurosomes were incubated with just 50 µCi S^35^-L-methionine. The samples were precipitated with equal volume of ice cold 10% (w/v) TCA. The pellets were washed with ice cold 5% (w/v) TCA extensively followed by ice cold methanol until there was no detectable radioactivity in the washed solution. The washed pellets were resuspended in 0.1 N NaOH (Sodium Hydroxide) for counting in liquid scintillation.

### Electron microscopy for synaptoneurosomes

Synaptoneurosomes, prepared according to above mentioned protocol were examined using transmission electron microscopy. Synaptoneurosomes were fixed in 3% glutaraldehyde (v/v) and stored at 4 °C for 24 h. Sample was subsequently washed with phosphate buffered saline (10 mM, pH 7.4, PBS) and fixed in 1% osmium tetroxide for 90 min. Further dehydration was done with different grades of alcohol and propylene oxide. The sample was impregnated with 1:1 ratio of propylene oxide: araldite resin, which was increased up to 1:3 ratio followed by pure araldite resin for 3 h. Finally, the samples were embedded in flat mould and kept at 60 °C for 48 h for polymerization. Sections were taken on a Leica ultamicrotome and transferred on to copper grid. Characterization of the ultrastructure of the synaptoneurosomes was performed using the transmission electron microscope (Tecanai, USA)^38^.

### Immunoblotting

Animals were sacrificed after 7 days or 30 days of ET-1 injections, brains were removed hippocampus was dissected out and flash frozen in liquid nitrogen. The tissue was homogenized in 0.1 M potassium phosphate buffer (pH 7.4) containing 0.25 M sucrose, protease inhibitor mixture and phosphatase inhibitors. The brain homogenate was centrifuged at 1000×g for 10 min to obtain post nuclear supernatant. The protein concentration was measured by dye binding method using kit from Thermo scientific. The postnuclear fraction was resolved on 8–12% SDS/PAGE and transferred onto PVDF membrane. The membranes were probed with following primary antibodies to Iba-1, pAkt1 (Ser473, Thr308), Akt1, pGSK3β (Ser9), pmTOR (Ser2448), mTOR, pS6K (Thr389), S6K, p4EBP1 (Thr46/47) 4EBP1and LC3b I/II. Blots were normalized to β-tubulin. The bands were detected using chemiluminesence detection system (Bio-Rad Chemidoc-XRS) and analyzed with Imagelab software (Bio-Rad).

### Statistics

The data is expressed as mean ± standard deviation. The difference in mean is determined by analyzing two groups with two tailed parametric *t*-test assuming Gaussian distribution, *p* < 0.05 was considered as significant in all the analysis.

## Supplementary Information

Supplementary Figures.

## References

[1] Sweeney MD, Sagare AP, Zlokovic BV (2018). Blood–brain barrier breakdown in Alzheimer disease and other neurodegenerative disorders. Nat Rev. Neurol..

[2] Kalaria RN (2018). The pathology and pathophysiology of vascular dementia. Neuropharmacology.

[3] Wardlaw JM, Smith C, Dichgans M (2013). Mechanisms of sporadic cerebral small vessel disease: insights from neuroimaging. Lancet Neurol..

[4] Arvanitakis Z, Leurgans SE, Barnes LL, Bennett DA, Schneider JA (2011). Microinfarct pathology, dementia, and cognitive systems. Stroke.

[5] Frey BM, Petersen M, Mayer C, Schulz M, Cheng B, Thomalla G (2019). Characterization of white matter hyperintensities in large-scale MRI-studies. Front. Neurol..

[6] Kowalczyk A, Kleniewska P, Kolodziejczyk M, Skibska B, Goraca A (2015). The role of Endothelin-1 and Endothelin receptor antagonists in inflammatory response and sepsis. Arch. Immunol. Ther. Exp. (Warsz).

[7] Hung VKL (2015). Selective astrocytic Endothelin-1 overexpression contributes to dementia associated with ischemic stroke by exaggerating astrocyte-derived amyloid secretion. J. Cereb. Blood Flow Metab..

[8] McCabe C, Arroja MM, Reid EI, Macrae M (2018). Animal models of ischaemic stroke and characterisation of the ischaemic penumbra. Neuropharmacology.

[9] Ansari S (2013). Endothelin-1 induced middle cerebral artery occlusion model for Ischemic stroke with laser Doppler flowmetry guidance in rat. J. Vis. Exp..

[10] Ahmad F (2017). Reactive oxygen species-mediated loss of synaptic Akt1 signaling leads to deficient activity-dependent protein translation early in Alzheimer’s disease. Antioxidants Redox Signal..

[11] Jung CH, Ro SH, Cao J, Otto NM, Kim DH (2010). mTOR regulation of autophagy. FEBS Lett..

[12] Davenport AP (2016). Endothelin. Barker EL, editor. Pharmacol. Rev..

[13] Sheng T, Zhang X, Wang S, Zhang J, Lu W, Dai Y (2015). Endothelin-1-induced mini-stroke in the dorsal hippocampus or lateral amygdala results in deficits in learning and memory. J. Biomed. Res..

[14] Tennant KA, Jones TA (2009). Sensorimotor behavioral effects of endothelin-1 induced small cortical infarcts in C57BL/6 mice. J. Neurosci. Methods.

[15] Windle V (2006). An analysis of four different methods of producing focal cerebral ischemia with endothelin-1 in the rat. Exp. Neurol..

[16] Wang Y, Jin K, Greenberg DA (2007). Neurogenesis associated with endothelin-induced cortical infarction in the mouse. Brain Res..

[17] Gross PM, Wainman DS, Espinosa FJ, Nag S, Weaver DF (1992). Cerebral hypermetabolism produced by intraventricular endothelin-1 in rats: inhibition by nimodipine. Neuropeptides.

[18] Horie N, Maag AL, Hamilton SA, Shichinohe H, Bliss TM, Steinberg GK (2008). Mouse model of focal cerebral ischemia using endothelin 1. J. Neurosci. Methods.

[19] Rennels ML, Gregory TF, Blaumanis OR, Fujimoto K, Grady PA (1985). Evidence for a ‘paravascular’ fluid circulation in the mammalian central nervous system, provided by the rapid distribution of tracer protein throughout the brain from the subarachnoid space. Brain Res..

[20] Brinker T, Stopa E, Morrison J, Klinge P (2014). A new look at cerebrospinal fluid circulation. Fluids Barriers CNS.

[21] de Lange EC (2004). Potential role of ABC transporters as a detoxification system at the blood-CSF barrier. Adv Drug Deliv. Rev..

[22] Strazielle N, Ghersi-Egea JF (2000). Choroid plexus in the central nervous system: biology and physiopathology. J. Neuropathol. Exp. Neurol..

[23] Bedussi B (2015). Clearance from the mouse brain by convection of interstitial fluid towards the ventricular system. Fluids Barriers CNS.

[24] Deane R (2003). RAGE mediates amyloid-beta peptide transport across the blood-brain barrier and accumulation in brain. Nat. Med..

[25] Deane R (2012). A multimodal RAGE-specific inhibitor reduces amyloid β-mediated brain disorder in a mouse model of Alzheimer disease. J. Clin. Investig..

[26] Zlokovic BV (2008). The blood-brain barrier in health and chronic neurodegenerative disorders. Neuron.

[27] Zlokovic BV (2005). Neurovascular mechanisms of Alzheimer’s neurodegeneration. Trends Neurosci..

[28] Zlokovic BV (2011). Neurovascular pathways to neurodegeneration in Alzheimer’s disease and other disorders. Nat. Rev. Neurosci..

[29] Nikolakopoulou AM (2019). Pericyte loss leads to circulatory failure and pleiotrophin depletion causing neuron loss. Nat. Neurosci..

[30] Wardlaw JM, Smith C, Dichgans M (2019). Small vessel disease: mechanisms and clinical implications. Lancet Neurol..

[31] Sehgal N (2012). Withania somnifera reverses Alzheimer’s disease pathology by enhancing low-density lipoprotein receptor-related protein in liver. Proc. Natl. Acad. Sci. USA.

[32] Montagne A (2018). Pericyte degeneration causes white matter dysfunction in the mouse central nervous system. Nat. Med..

[33] Sarnat HB, Nochlin D, Born DE (1998). Neuronal nuclear antigen (NeuN): a marker of neuronal maturation in early human fetal nervous system. Brain Dev..

[34] Vorhees CV, Williams MT (2006). Morris water maze: procedures for assessing spatial and related forms of learning and memory. Nat. Protoc..

[35] Leger M (2013). Object recognition test in mice. Nat. Protoc..

[36] Fernagut PO, Diguet E, Labattu B, Tison F (2002). A simple method to measure stride length as an index of nigrostriatal dysfunction in mice. J. Neurosci. Methods.

[37] Muddashetty RS, Kelić S, Gross C, Xu M, Bassell GJ (2007). Dysregulated metabotropic glutamate receptor-dependent translation of AMPA receptor and postsynaptic density-95 mRNAs at synapses in a mouse model of fragile X syndrome. J. Neurosci..

[38] Ahmad F (2018). Isoform-specific hyperactivation of calpain-2 occurs presymptomatically at the synapse in Alzheimer's disease mice and correlates with memory deficits in human subjects. Sci. Rep..

