# Supplementary material for: Endothelin-1 mediated vasoconstriction leads to memory impairment and synaptic dysfunction
Source: Sci Rep. 2021 Mar 1;11:4868. doi: 10.1038/s41598-021-84258-x (PMC7921549; doi:10.1038/s41598-021-84258-x)

Endothelin-1 mediated vasoconstriction leads to memory impairment and synaptic dysfunction

Latha Diwakar[1], Ruturaj Gowaikar[1], Keerthana Chithanathan[1], Barathan Gnanabharathi[1], Deepika Singh Tomar[1], and Vijayalakshmi Ravindranath [1,2] *

[1]Centre for Neuroscience, Indian Institute of Science, Bangalore - 560012, India
[2]Centre for Brain Research, Bangalore – 560012, India

Supplementary Figure.1.

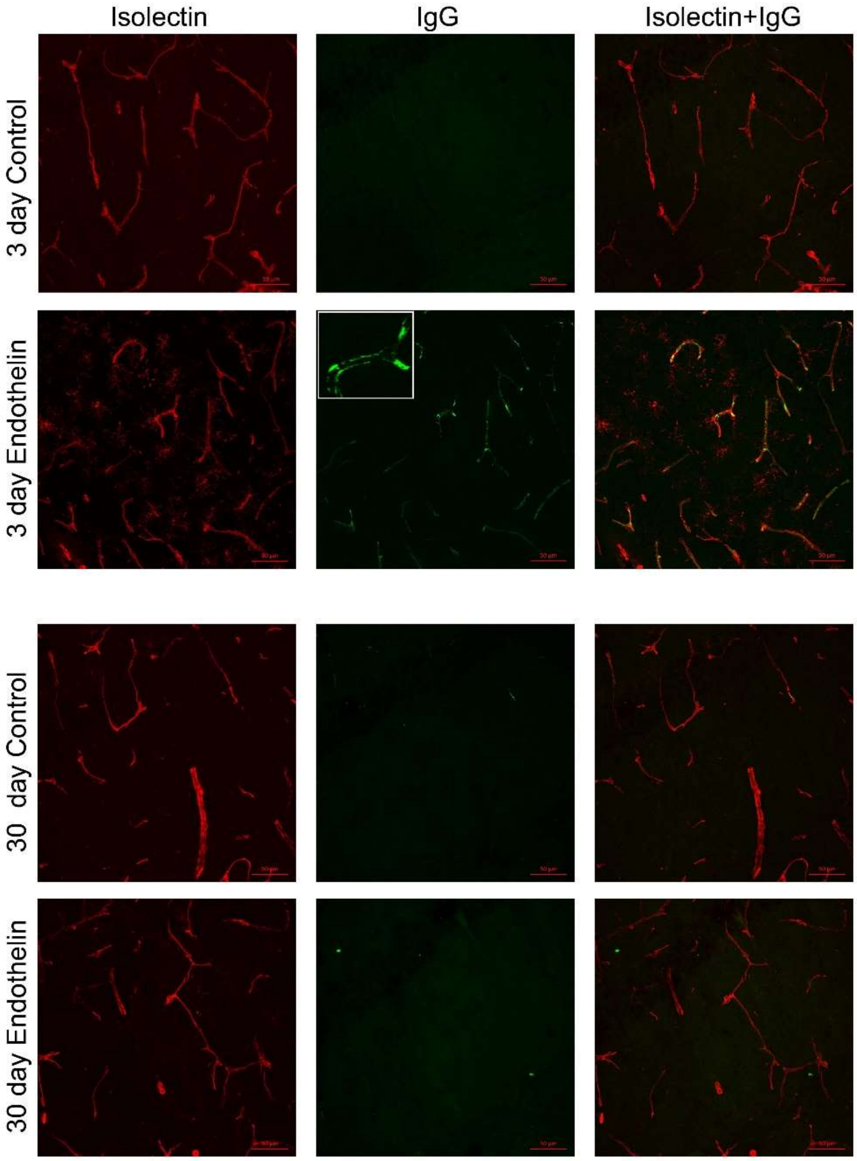

Supplementary Figure.1. Lectin (red) positive blood vessels and anti-IgG (green) staining for blood derived IgG during BBB leakage. There was lectin positive staining at all time points in ET-1 injected and control mice. There were deposits of IgG after 3 days of ET-1 injection, while there was no IgG staining in control indicating BBB leakage (inset showing IgG staing). However, there was no staining at 30 days of ET-1 treatment.

Supplementary Figure. 2.

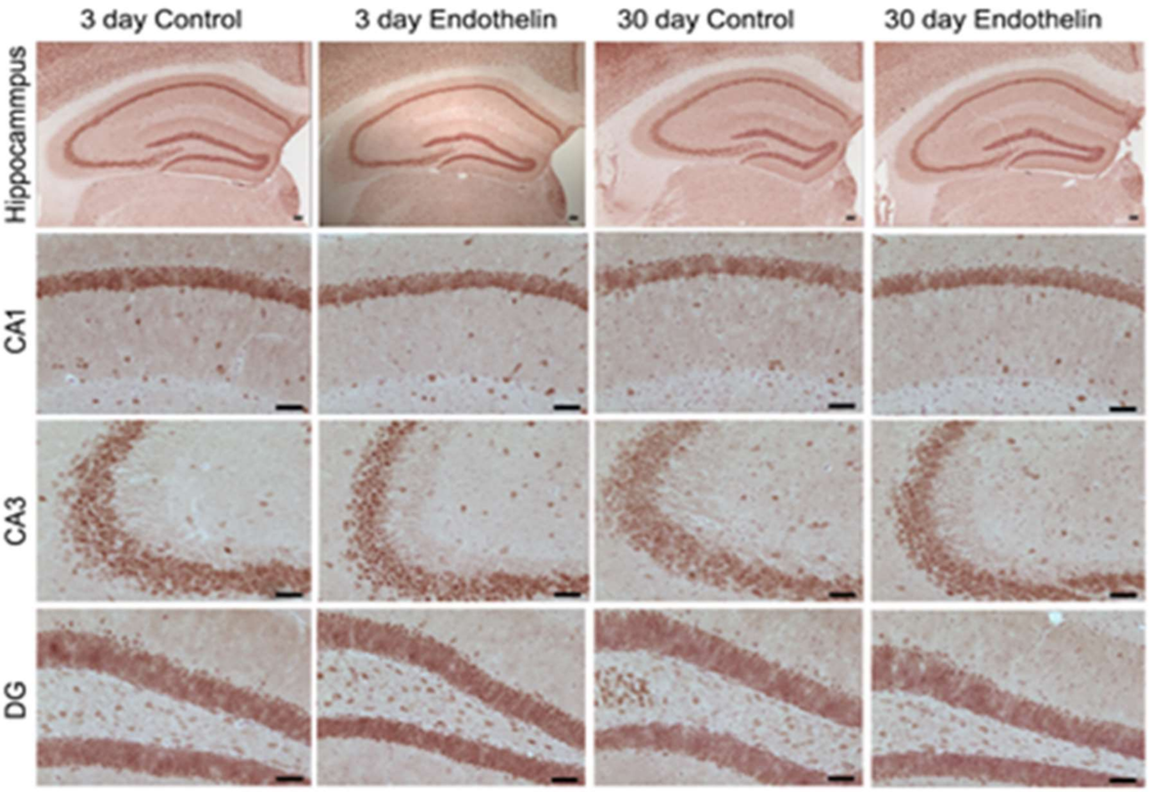

Supplementary Figure.2. NeuN immunohistochemistry in the hippocampus of the mice injected with ET-1. NeuN-immunoreactive neurons did not show any changes among the groups. First panel show 4X images of whole hippocampus from all the groups. Bottom panels show different regions of hippocampus at 20X. There was no neuronal loss at any time points after ET-1 injection compared to control.

Supplementary Figure.3.

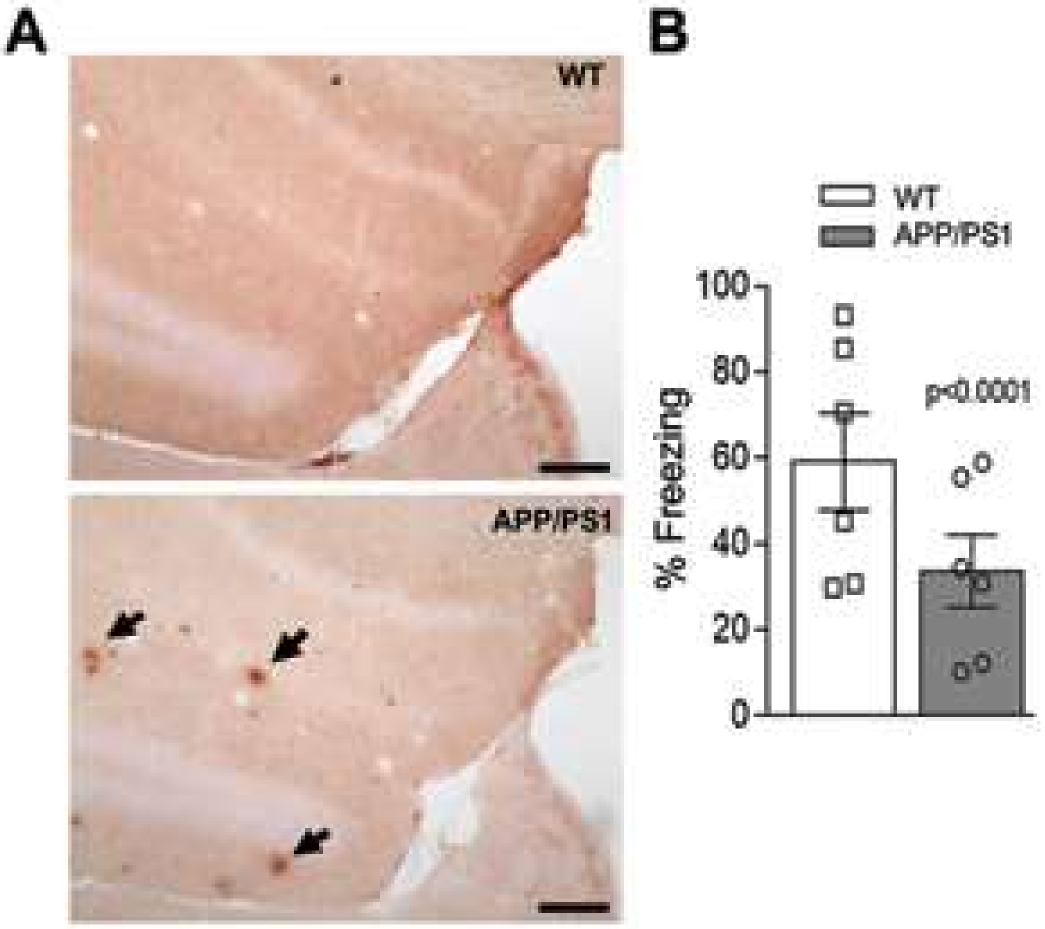

Supplementary Figure.3. Aβ plaques deposition and behavioral deficits observed after cFc test in 9 months old AD mice. **A.** Images show the immunohistochemical staining of plaque for Aβ antibody in 9 months old wild type and APP/PS1 mice in hippocampal region. **B.** There was decrease in percentage of freezing in AD mice after shock at day one and recall done after 24 h. Values are expressed as Mean ± SD in seconds for n=6 animals.

Supplementary Figure.4.

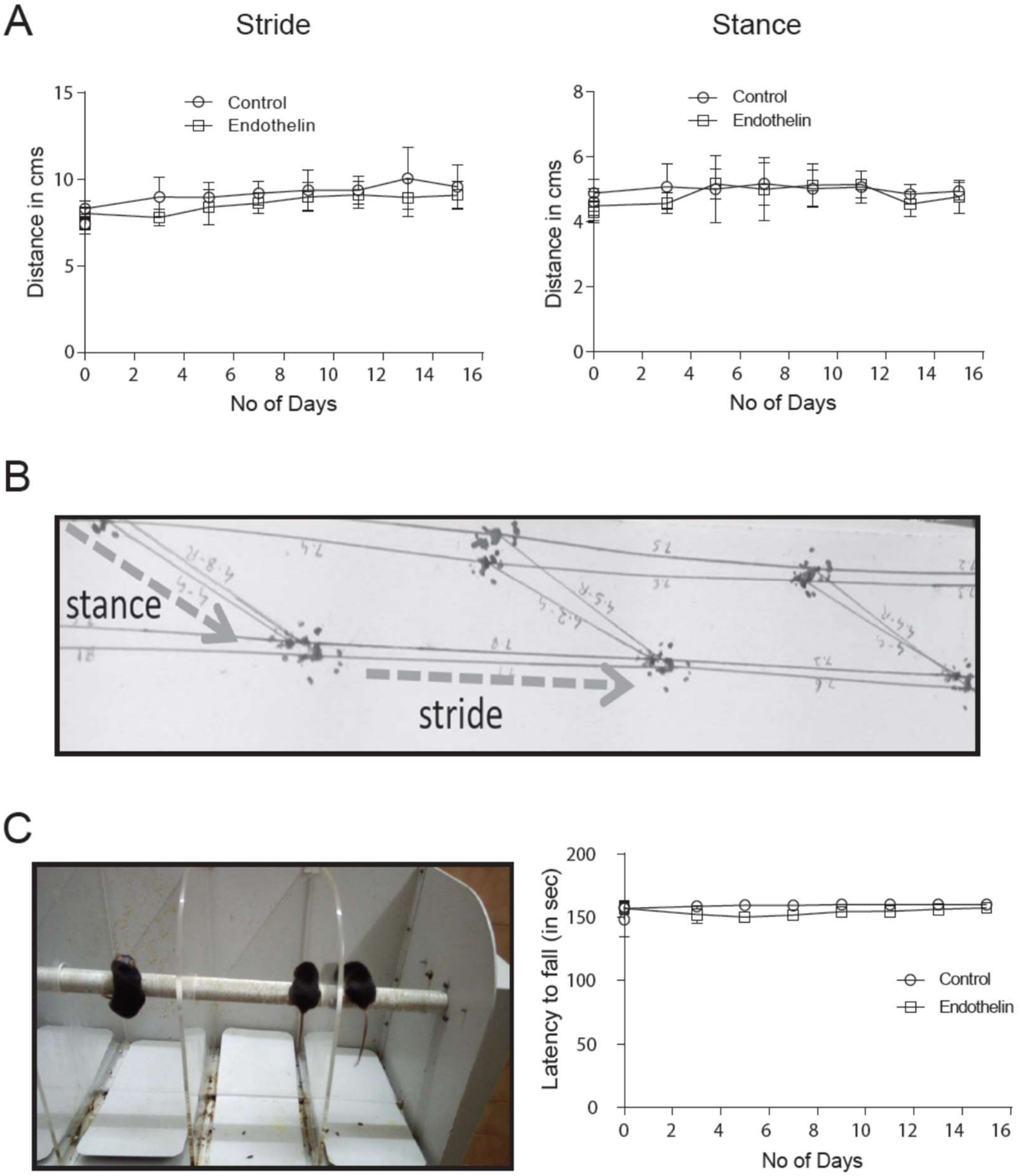

Supplementary Figure.4. **Analysis of footprints and Rotarod balancing for 15 days after ET-1 injection in C57 mice**. **A.** Graph shows the stride and stance length measured in centimeters for 15 days of endothelin treatment. The dashed box depicts the transient increase in stance length (Day3-7) in the mice upon endothelin treatment. The values are mean length of n=8 animals however it was statistically not significant. **B.** Representative footprints to show the measurement of stride and stance length. **C.** Image of rotarod and representative graph to show the measurement of time in seconds the mice able to balance on the rotarod. The values are Mean ± SD of n=8 animals.

Supplementary Figure.5

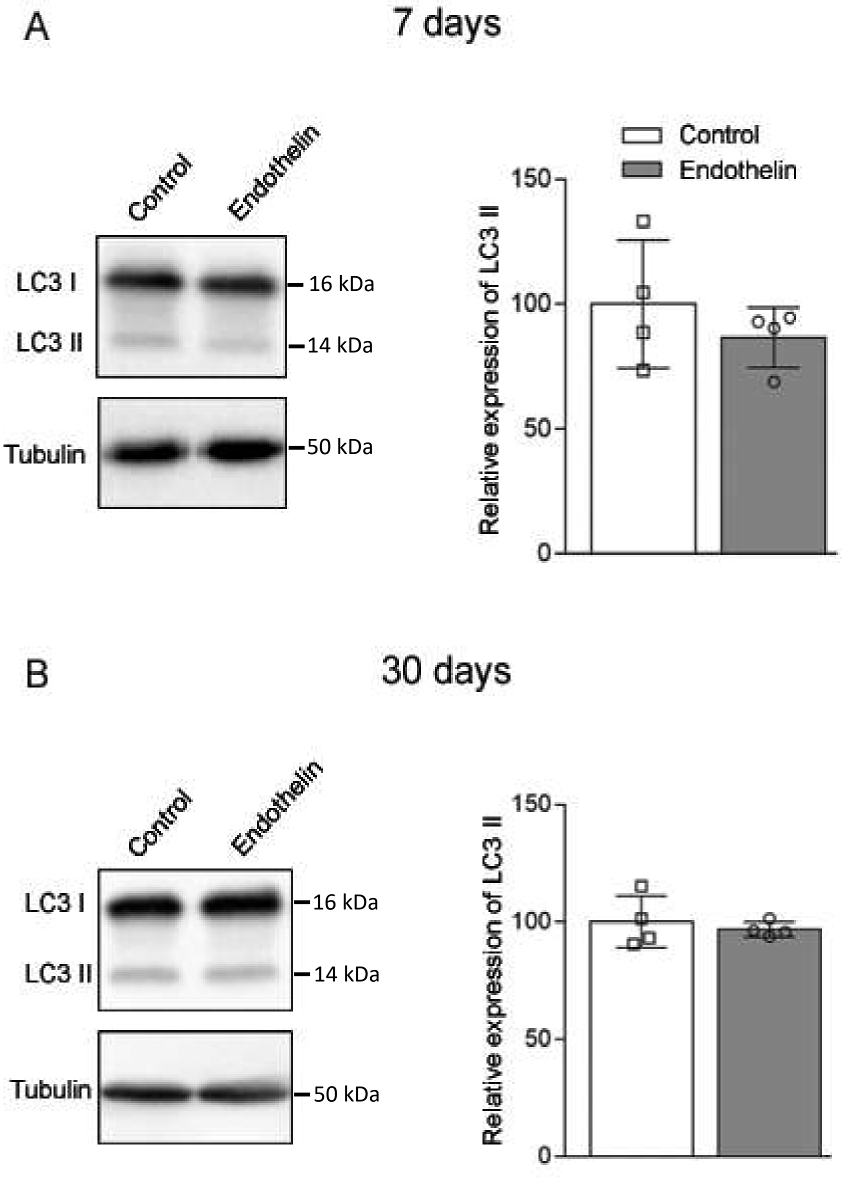

Supplementary Figure.5. Expression of LC3 I/II after 7 and 30 days of ET-1 treatment. A and B. The immunoblot for LC3 I/II expression in hippocampal lysate did not show any change after 7 or 30 days of injection indicating that ET-1 did not trigger autophagy. The graph shows the relative expression of LC3 II in ET-1 treated mice compared to vehicle controls (n=4). Data are represented as Mean ± SD (n = 4 mice).

Supplementary Figure.6

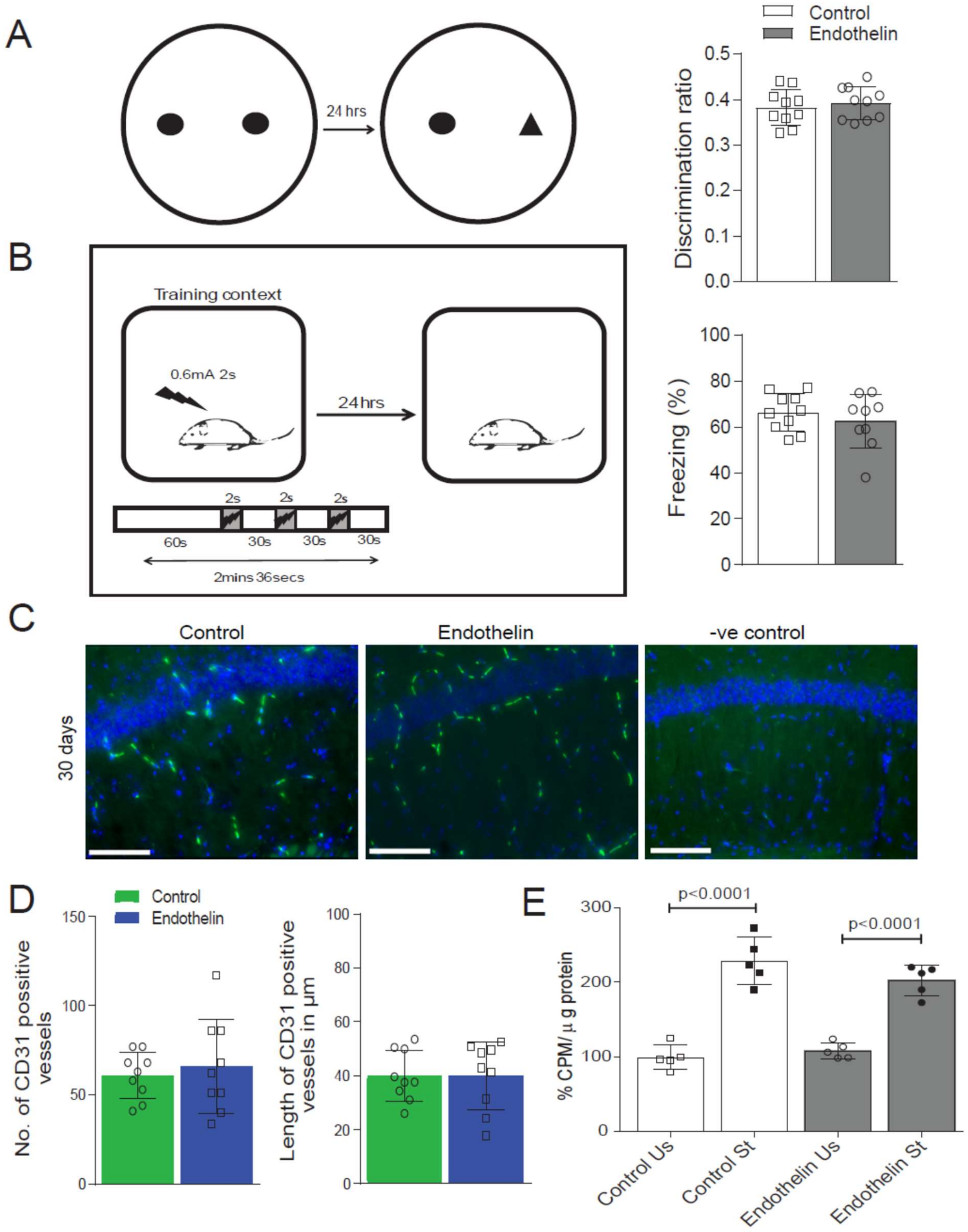

Supplementary Figure.6. **Reversal in memory deficit and CD31 expression by 30 days of ET-1 treatment. A.** Shows graphical representation of novel object paradigm and graph showed no difference in discrimination ratio in ET-1 injected mice after 30 days. The graph shows Mean ± SD of n=10 animals p=0.566. **B.** The percentage of freezing did not decrease when mice were given shock on 29th day and recall was after 30 days of ET-1 injection. p=0.4068. Values are expressed as Mean ± SD in seconds for n=10 animals. **C.** Representative images shows CD31 expression was reversed by 30 days of ET-1 treatment. **D.** The graphical representation of number and length of CD31 positive vessels from three sections of hippocampus of control and ET-1 injected mice expressed as Mean ± SD of n=3 animals. Statistically there was no difference in number and length of CD31 positive vessels between saline and ET- injected animals after 30 days (p=6536, p=4415). **E.** S^{35}-methionine incorporation was unaffected in synaptoneurosomes prepared from 30 days of ET-1 treatment after KCl stimulation. We saw stimulation both in control and ET-1 injected mice (p<0.0001). Data are represented as Mean ± SD (n = 5 mice).

Supplementary Figure.7

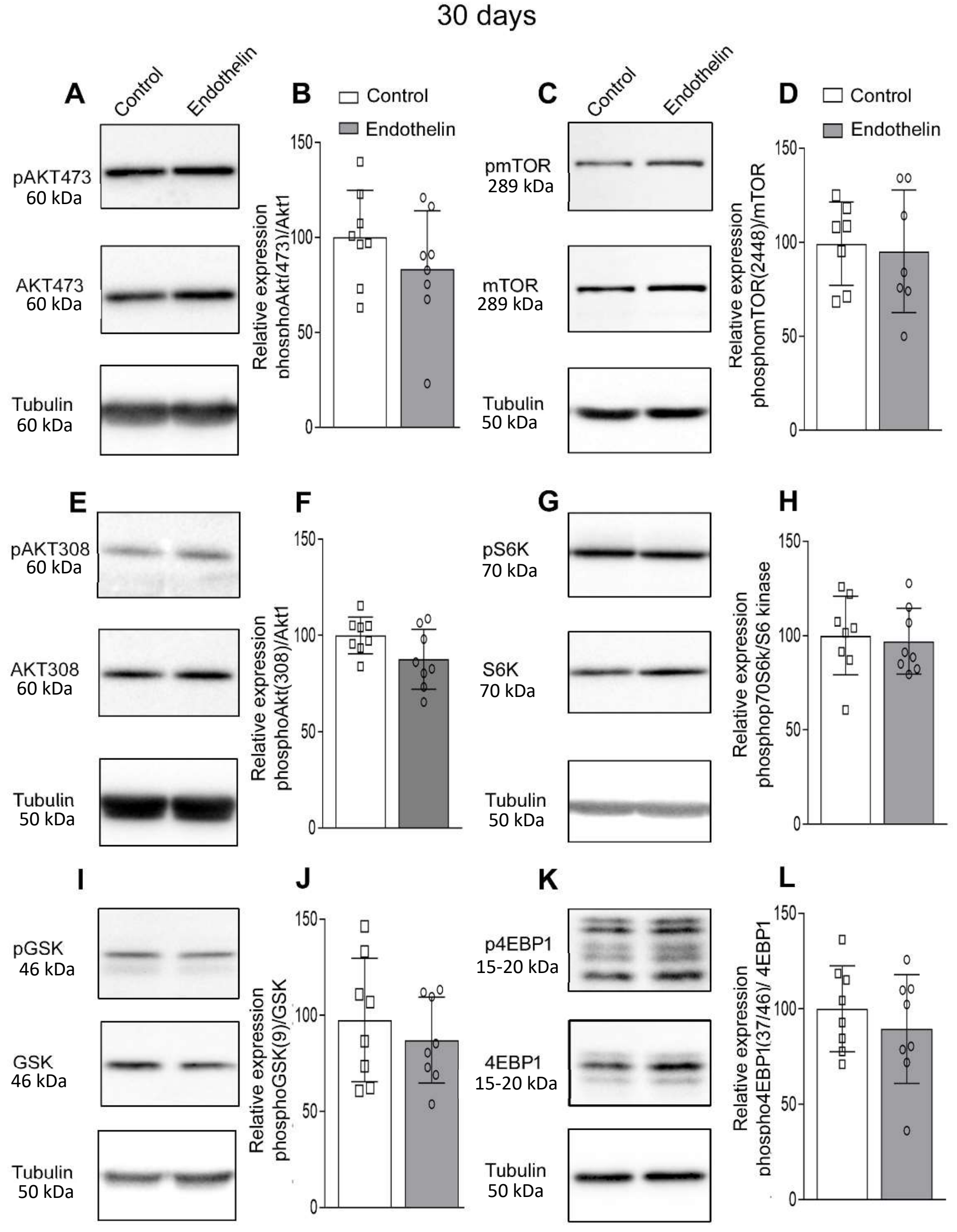

Supplementary Figure.7. Akt-mTOR phosphorylation after 30 days of ET-1 treatment. A, B, E, F, I and J. The levels of both phosphorylated forms of Akt1 Ser473 and Thr308 as well as GSK did not show any difference after 30 days of ET-1 treatment. Values are Mean ± SD, n=8 animals. **C, D, G, H, K and L.** There was no change in levels of phosphorylated mTOR and its downstream molecules p4EBP at Thr46/47 and pS6K at Thr389 in hippocampal lysate of bilateral ET-1 injected mice compared to controls after 30 days. Values are Mean ± SD, n=8 animals.

Supplementary Figure.8

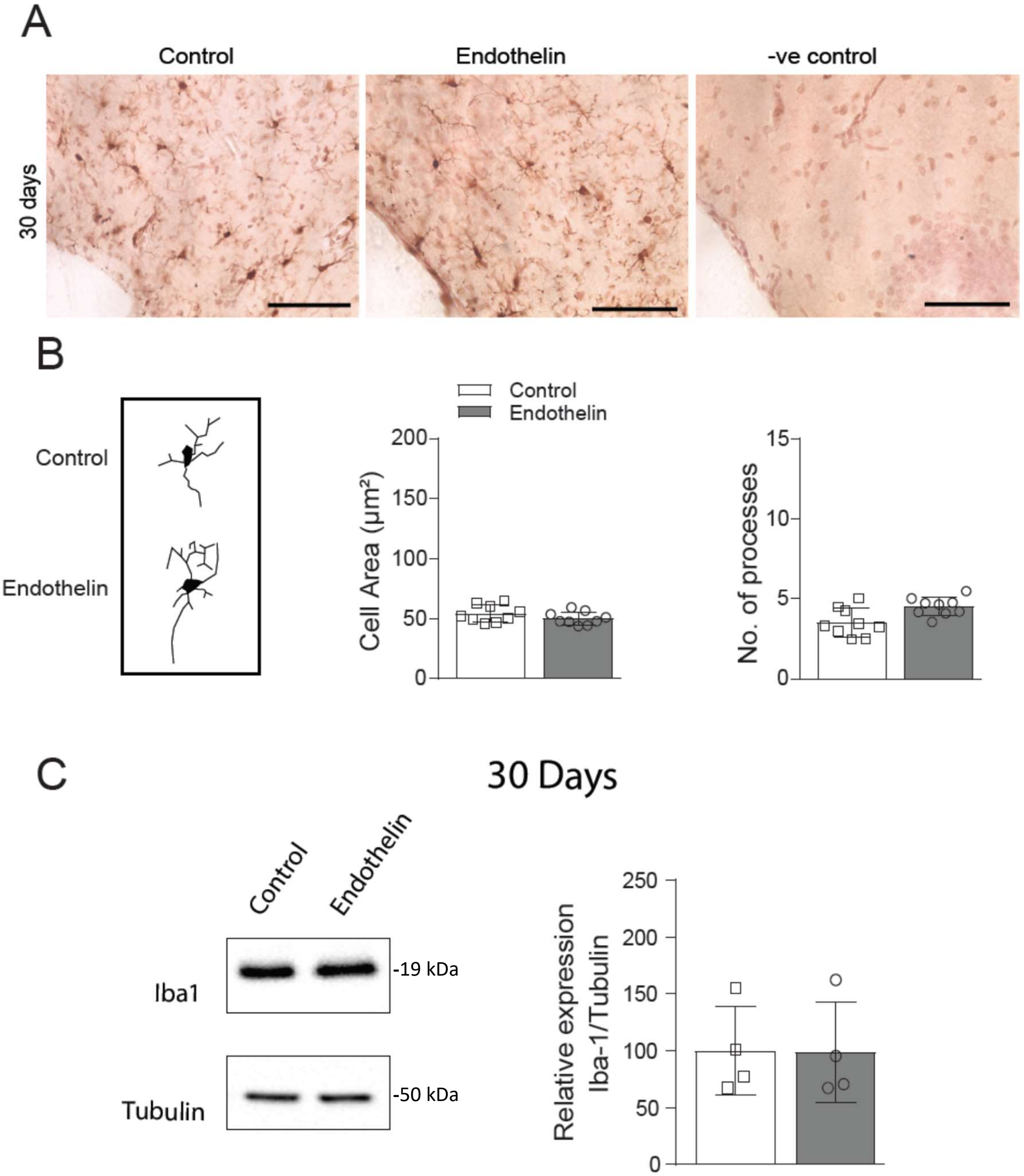

Supplementary Figure.8. Expression of Iba-1 showing microglia near the ventricles after 30 days of ET-1 treatment. A. ET-1 treatment did not stimulate microglial activation after 30 days around the ventricles. **B**. The graph shows no difference in cell area and number of process in microglia of ET-1 treated mice compared to vehicle controls (n=3). **C.** The immunoblot for Iba-1 expression in hippocampal lysate did not show any change after 30 days of ET-1 treatment. Data are represented as Mean ± SD (n = 4 mice).

Full blots for all the figures

Figure. 3 full-length blots

Iba-1-7 Days ET-1 treatment

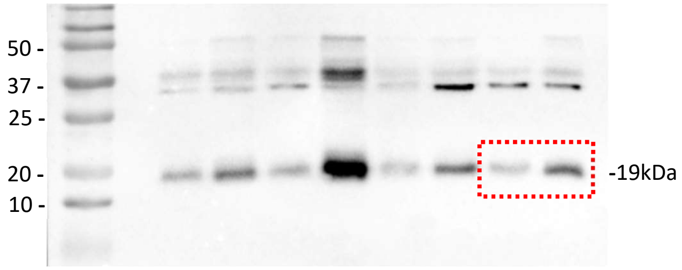

Tubulin for Iba-1

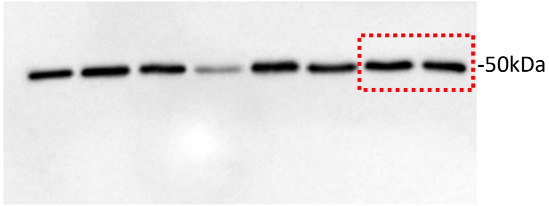

Figure. 5 full-length blots

pAKT1(473)- 7 Days ET-1 treatment

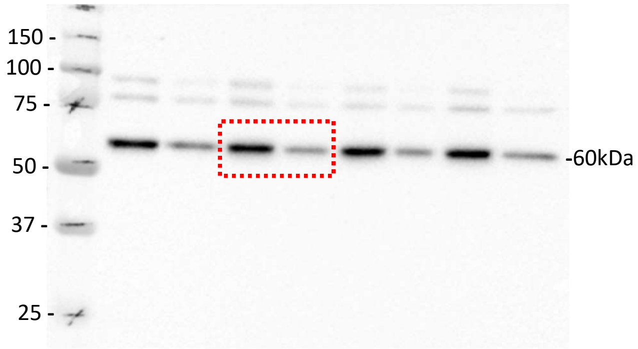

pmTOR- 7 Days ET-1 treatment

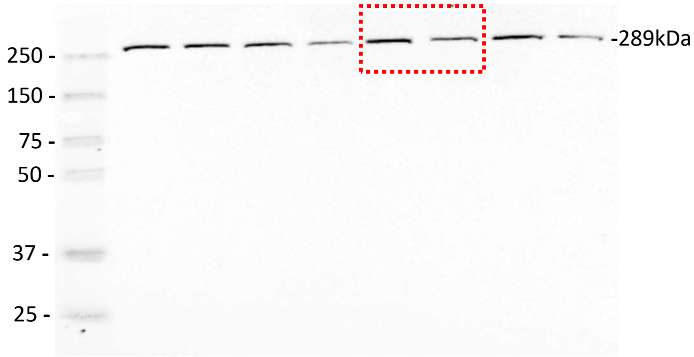

TAKT1(473)- 7 Days ET-1 treatment

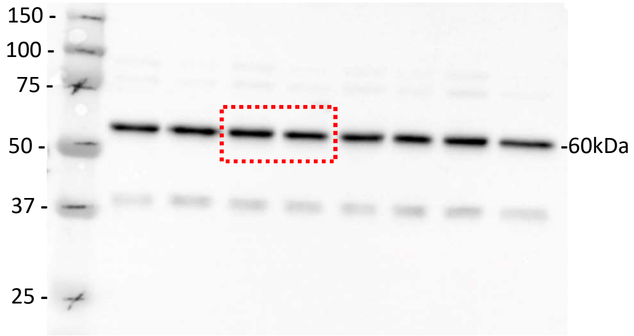

TmTOR- 7 Days ET-1 treatment

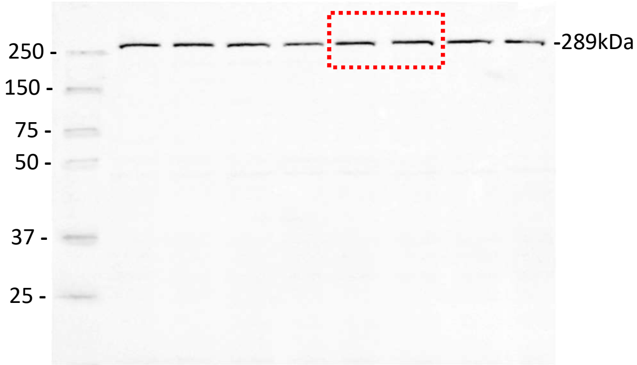

Tubulin for AKT (473)

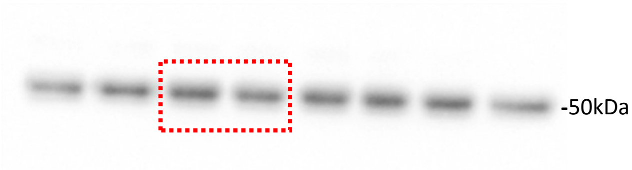

Tubulin for mTOR

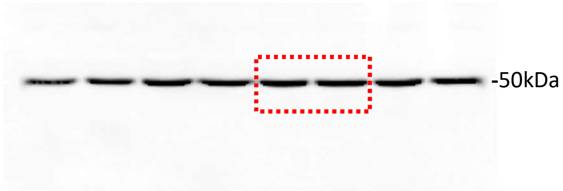

Figure. 5 full-length blots

pAKT1(308)- 7 Days ET-1 treatment

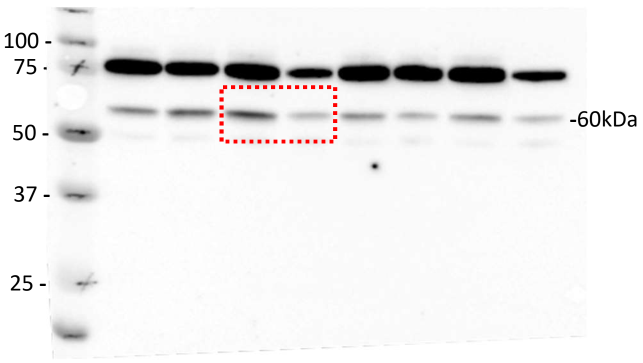

pS6K- 7 Days ET-1 treatment

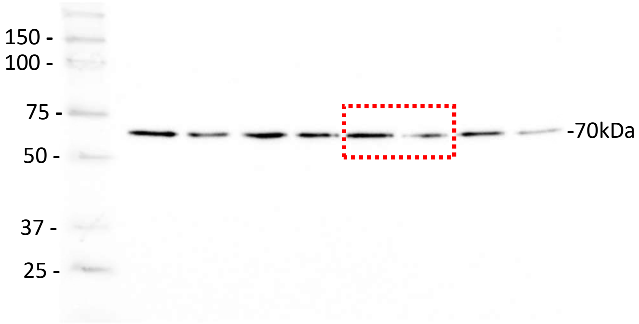

TAKT1(308)- 7 Days ET-1 treatment

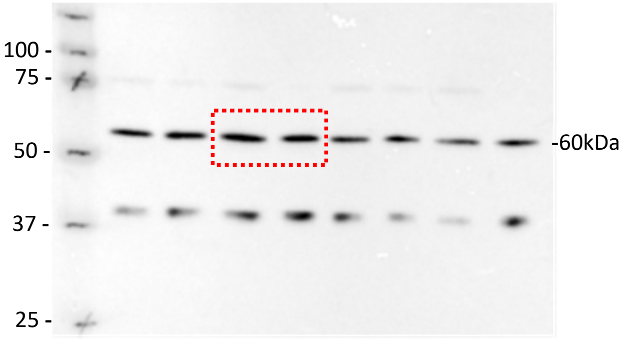

TS6K- 7 Days ET-1 treatment

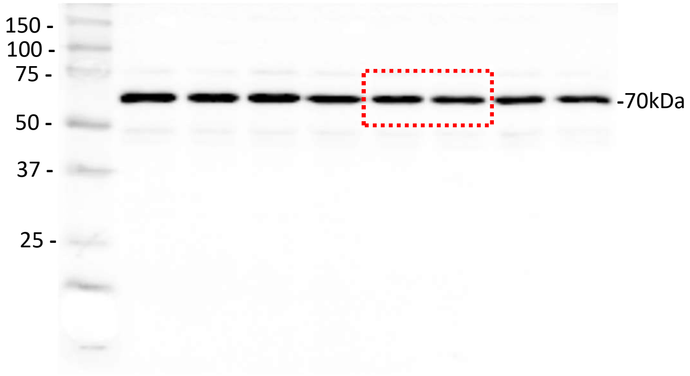

Tubulin for AKT

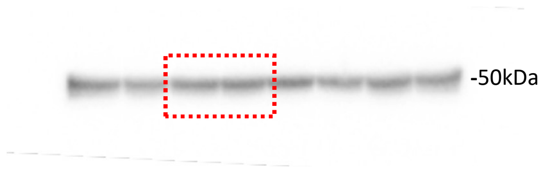

Tubulin for S6K

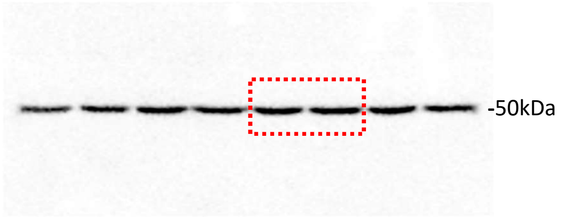

Figure. 5 full-length blots

pGSK- 7 Days ET-1 treatment

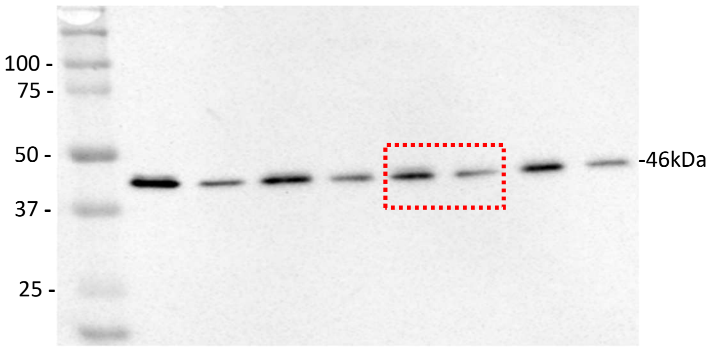

p4EBP1- 7 Days ET-1 treatment

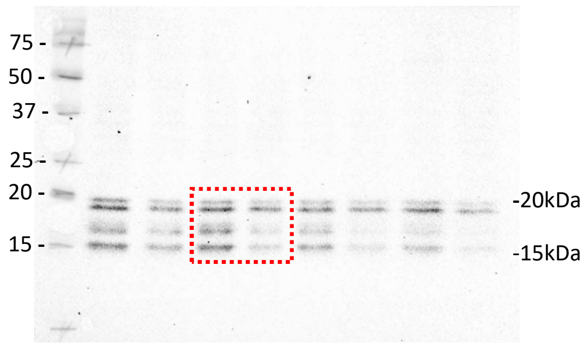

TGSK- 7 Days ET-1 treatment

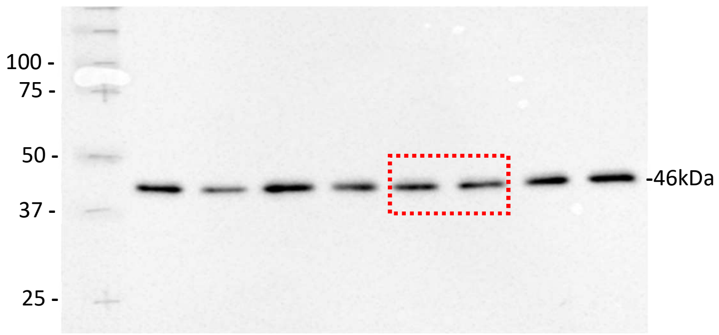

T4EBP1- 7 Days ET-1 treatment

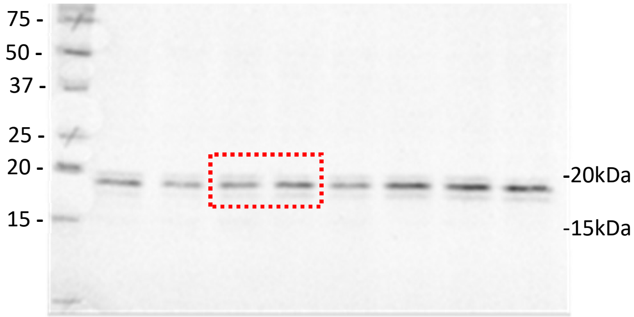

Tubulin for GSK

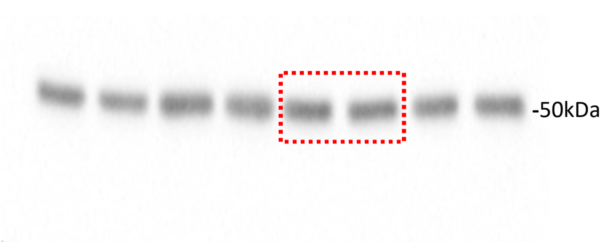

Tubulin for 4EBP1

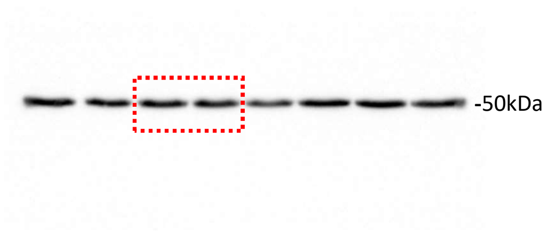

Supplementary Figure. 5 full-length blots

LC3 I/II - 7 Days ET-1 treatment

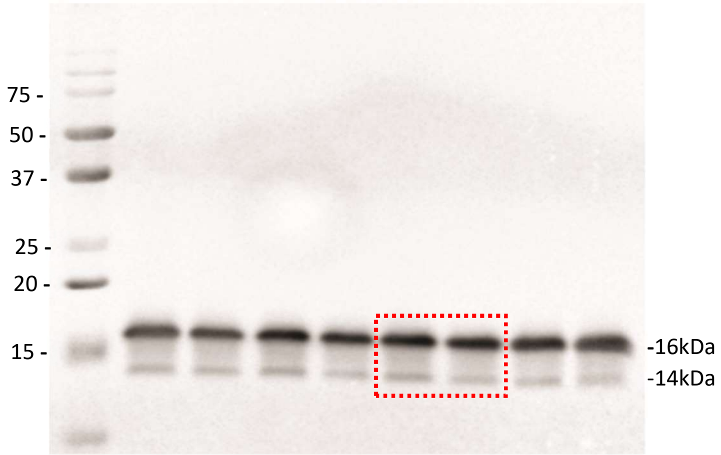

Tubulin for LC3 I/II - 7days

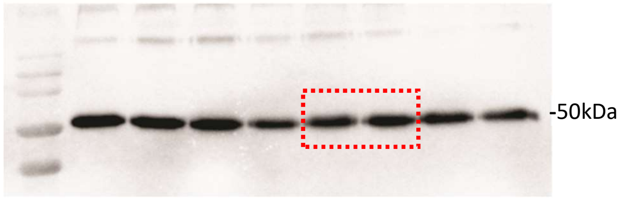

LC3 I/II - 30 Days ET-1 treatment

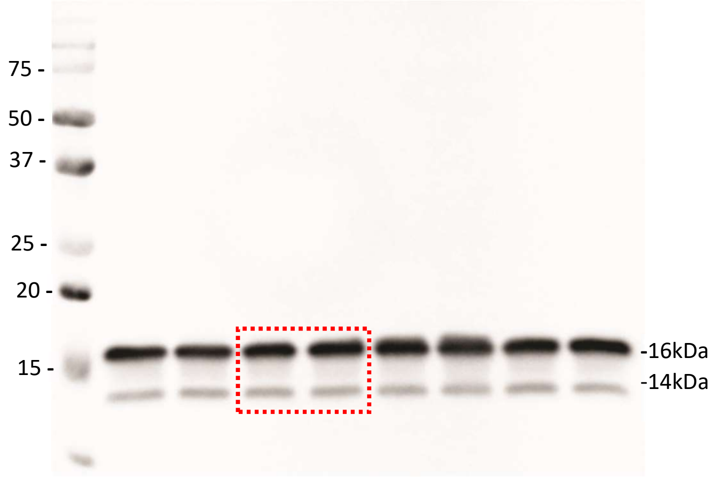

Tubulin for LC3 I/II - 30 days

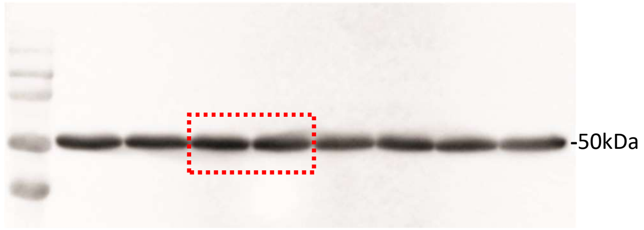

Supplementary Figure. 7 full-length blots

pAKT1(473)- 30 Days ET-1 treatment

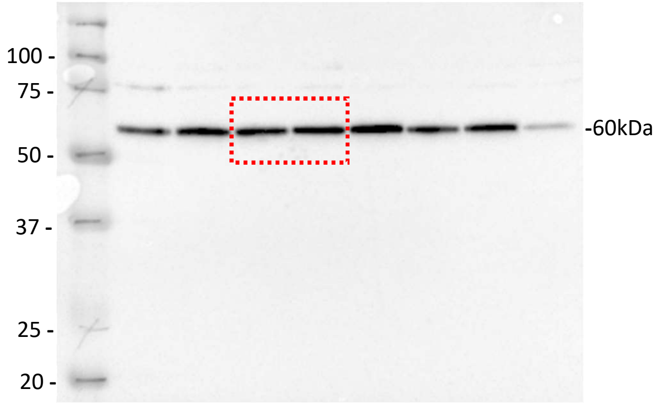

pmTOR- 30 Days ET-1 treatment

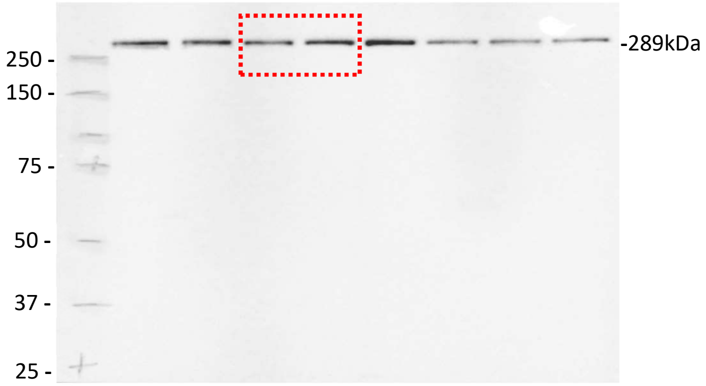

Supplementary Figure. 7 full-length blots

TAKT1(473)- 30 Days ET-1 treatment

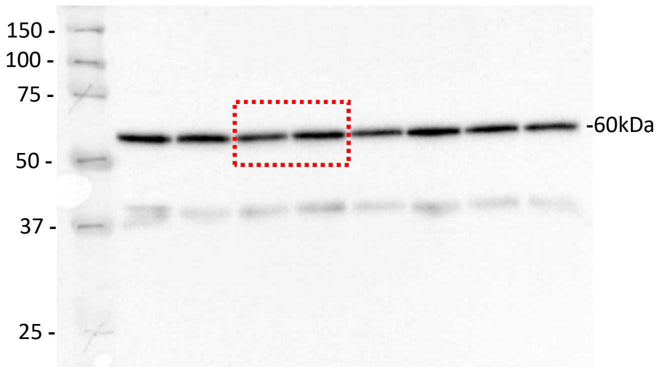

TmTOR- 30 Days ET-1 treatment

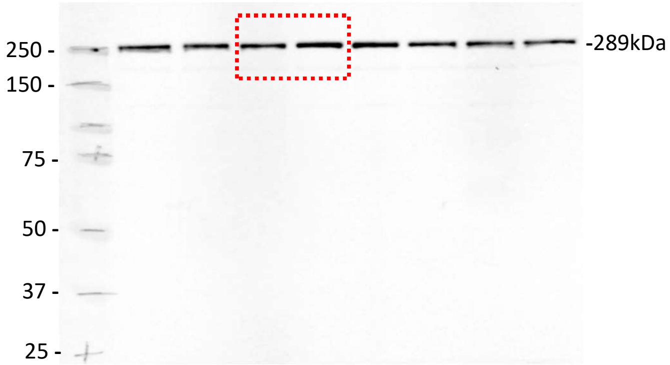

Tubulin for AKT 473

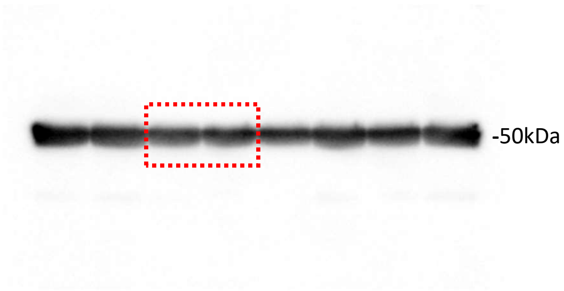

Tubulin for mTOR

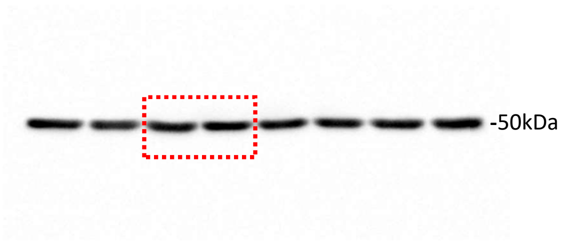

pAKT1(308)- 30 Days ET-1 treatment

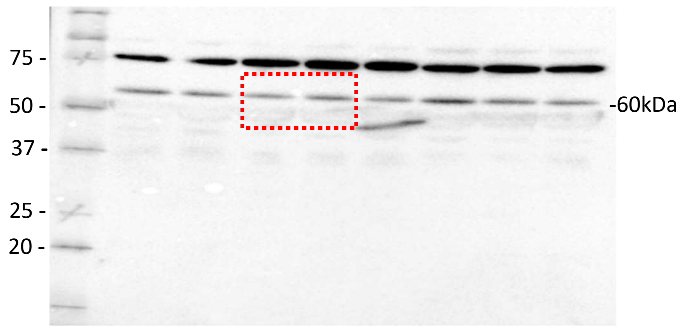

pS6K- 30 Days ET-1treatment

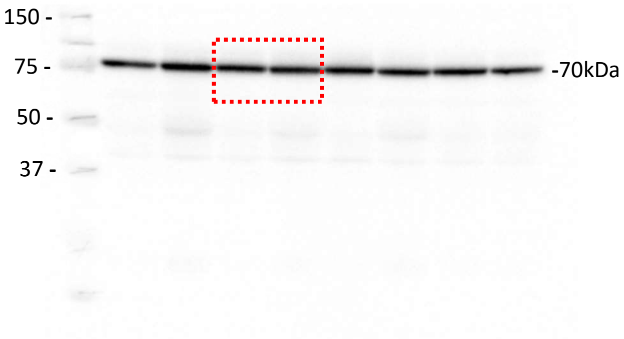

Supplementary Figure. 7 full-length blots

TAKT1(308)- 30 Days ET-1 treatment

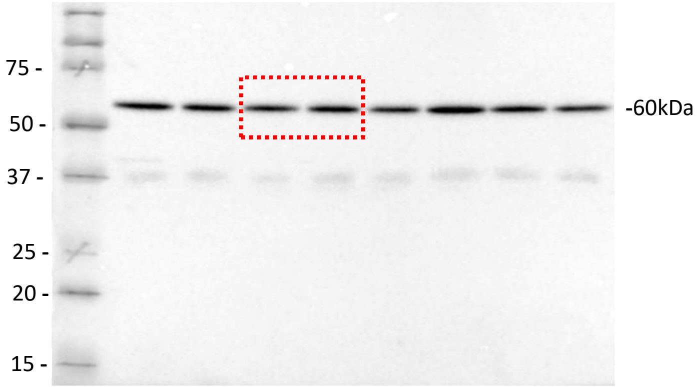

TS6K- 30 Days ET-1 treatment

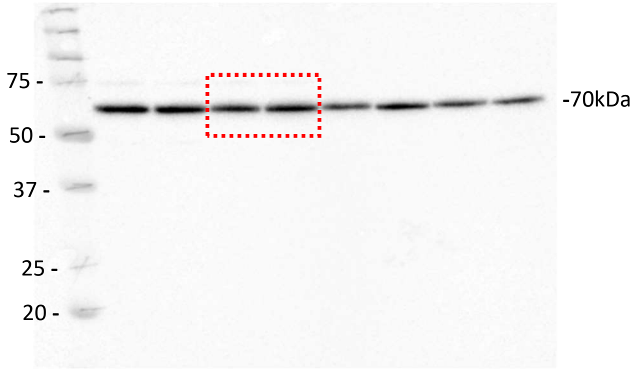

Tubulin for AKT 308

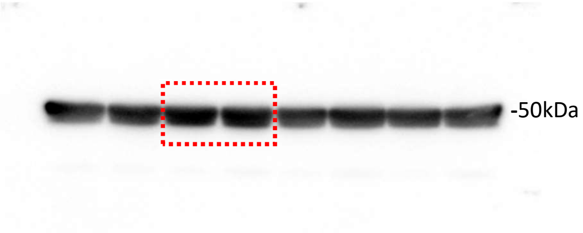

Tubulin for S6K

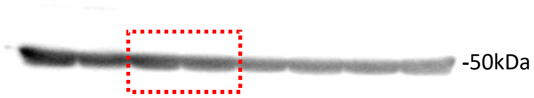

pGSK - 30 Days ET-1 treatment

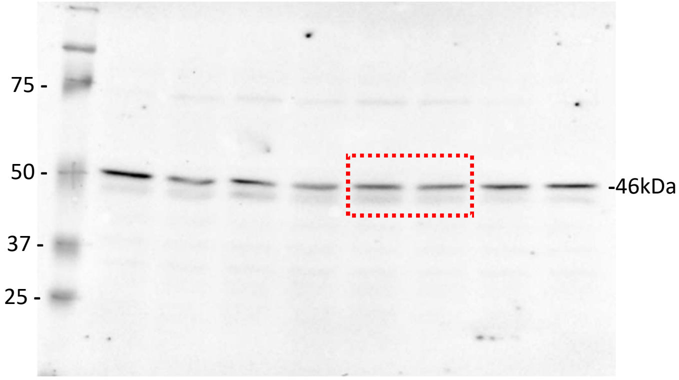

p4EBP1- 30 Days ET-1treatment

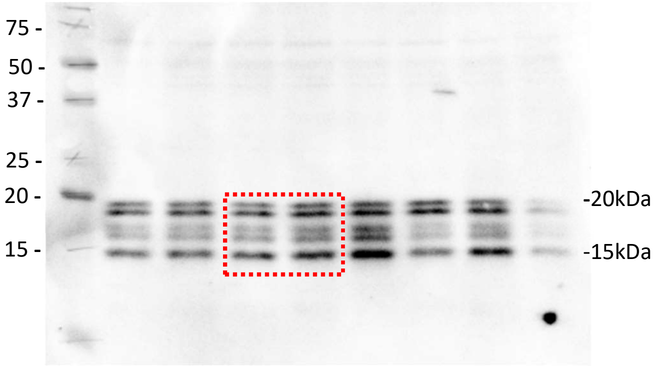

Supplementary Figure. 7 full-length blots

TGSK - 30 Days ET-1 treatment

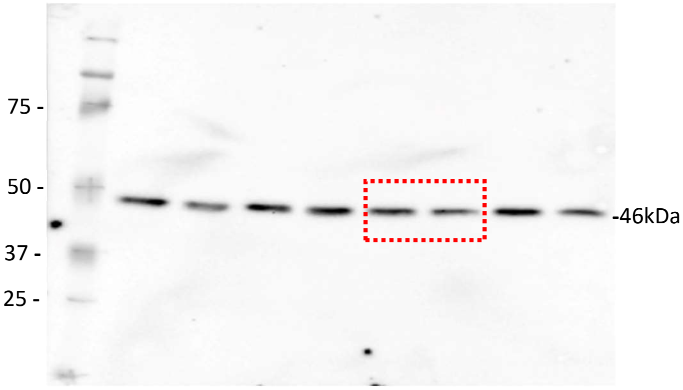

T4EBP1- 30 Days ET-1treatment

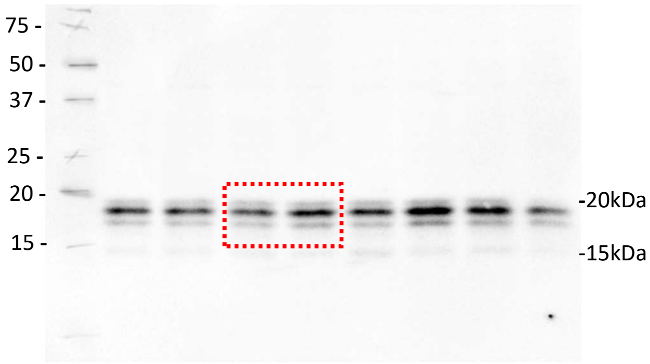

Tubulin for GSK

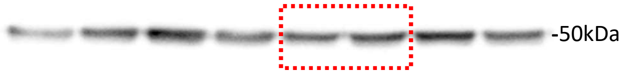

Tubulin for 4EBP1

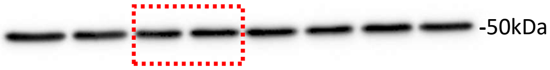

Supplementary Figure. 8 full-length blots

Iba-1-30 Days ET-1 treatment

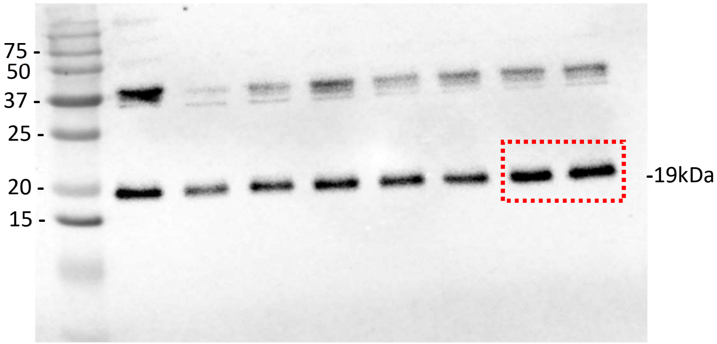

Tubulin for Iba-1

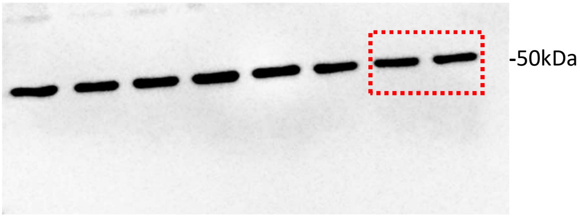

Supplement: Supplementary file 1 — Supplementary Figures. [file 41598_2021_84258_MOESM1_ESM.pdf]
